# How do “robopets” impact the health and well‐being of residents in care homes? A systematic review of qualitative and quantitative evidence

**DOI:** 10.1111/opn.12239

**Published:** 2019-05-09

**Authors:** Rebecca Abbott, Noreen Orr, Paige McGill, Rebecca Whear, Alison Bethel, Ruth Garside, Ken Stein, Jo Thompson‐Coon

**Affiliations:** ^1^ Evidence Synthesis Team, NIHR CLAHRC South West Peninsula (PenCLAHRC), College of Medicine and Health University of Exeter Exeter UK; ^2^ College of Medicine and Health University of Exeter Exeter UK; ^3^ European Centre for Environment and Human Health University of Exeter Medical School, University of Exeter Exeter UK

**Keywords:** Companion animals, dementia, long‐term care, older adults, robopets, social robots, systematic review

## Abstract

**Background:**

Robopets are small animal‐like robots which have the appearance and behavioural characteristics of pets.

**Objective:**

To bring together the evidence of the experiences of staff, residents and family members of interacting with robopets and the effects of robopets on the health and well‐being of older people living in care homes.

**Design:**

Systematic review of qualitative and quantitative research.

**Data sources:**

We searched 13 electronic databases from inception to July 2018 and undertook forward and backward citation chasing.

**Methods:**

Eligible studies reported the views and experiences of robopets from residents, family members and staff (qualitative studies using recognised methods of qualitative data collection and analysis) and the effects of robopets on the health and well‐being of care home residents (randomised controlled trials, randomised crossover trials and cluster randomised trials). Study selection was undertaken independently by two reviewers. We used the Wallace criteria and the Cochrane Risk of Bias tool to assess the quality of the evidence. We developed a logic model with stakeholders and used this as a framework to guide data extraction and synthesis. Where appropriate, we used meta‐analysis to combine effect estimates from quantitative studies.

**Results:**

Nineteen studies (10 qualitative, 2 mixed methods and 7 randomised trials) met the inclusion criteria. Interactions with robopets were described as having a positive impact on aspects of well‐being including loneliness, depression and quality of life by residents and staff, although there was no corresponding statistically significant evidence from meta‐analysis for these outcomes. Meta‐analysis showed evidence of a reduction in agitation with the robopet “Paro” compared to control (−0.32 [95% CI −0.61 to −0.04, *p* = 0.03]). Not everyone had a positive experience of robopets.

**Conclusions:**

Engagement with robopets appears to have beneficial effects on the health and well‐being of older adults living in care homes, but not all chose to engage. Whether the benefits can be sustained are yet to be investigated.

**Implications for practice:**

Robopets have the potential to benefit people living in care homes, through increasing engagement and interaction. With the robopet acting as a catalyst, this engagement and interaction may afford comfort and help reduce agitation and loneliness.

**What does this research add to existing knowledge in gerontology?**

- This is the first systematic review to synthesise research that has focussed on the experiences and effects of pet‐ or animal‐like robots (robopets) in older adult residential care settings.
- For those that choose to engage with them, robopets have the potential to reduce loneliness and agitation, increase social interactions and provide comfort and pleasure.
- Not everyone engages with robopets, and some older adults, families and nursing staff might actively dislike them.

**What are the implications of this new knowledge for nursing care with older people?**

- Training in how to best use and introduce robopets may help improve resident engagement and staff confidence in using them.
- Resident‐robopet interactions are highly varied and influenced by personal histories and the type and characteristics of the robopet.
- Whilst robopets should not be considered a replacement for human interaction, there appears to be scope for using them as therapy for agitated or isolated residents

**How could the findings be used to influence policy or practice or research or education?**

- Researchers can build on the gaps (shown in our final logic model) and use more appropriate outcome measures in future trials of robopets that assess staff, family and carer perspectives such as comfort, pleasure, appreciation and communication.
- There is a still a paucity of evidence on the long‐term sustainability of robopets: Does the novelty wear off or do interactions deepen?
- Until recently, robopets have been prohibitively expensive. A new wave of cheaper robopets may facilitate more robust research in this area.

## 1 BACKGROUND

In recent years, there has been increasing interest in the use of pet or animal‐assisted therapy in care and nursing homes as a type of non‐pharmacological therapy that can provide sensory enhancement and facilitate social contact (Bernabei et al., 2013; Eachus, 2001; Odendaal, 2000; Virues‐Ortega, Pastor‐Barriuso, Castellote, Poblacion, & de Pedro‐Cuesta, 2012). Research assessing the impact of animals on the health and well‐being of older people in residential care, including persons with dementia, has shown positive benefits in terms of companionship and engagement, along with reductions in depression and improvements in behavioural problems (Filan & Llewellyn‐Jones, 2006; Richeson, 2003; Virues‐Ortega et al., 2012). However, due to concerns regarding hygiene and safety, the limited availability of appropriate animals and the inability of care homes to meet the needs of living animals, pet therapy may not always be a suitable or viable option. Robotic animals that mimic living animals and respond to human interaction may offer an alternative therapy.

Robopets, a term first coined by Eachus in 2001, are small animal‐like robots which have the appearance and behavioural characteristics of companion animals or pets (Eachus, 2001). Examples of robopets reported in the literature include a baby harp seal (PARO), a robotic cat (NeCoRo) and a robotic dog (AIBO) (Preuss & Legal, 2017). Robopets fall under the broader umbrella of socially assistive companion robots, whose use in older adult care has been widely reviewed, but mostly from a quantitative perspective (Bemelmans, Gelderblom, Jonker, & De Witte, 2012; Mordoch, Osterreicher, Guse, Roger, & Thompson, 2013; Pu, Moyle, Jones, & Todorovic, 2019) and often across a broad base of care settings, not specifically residential care (Kachouie, Sedighadeli, Khosla, & Chu, 2014; Vandemeulebroucke, de Casterle, & Gastmans, 2018). Furthermore, the qualitative evidence around companion robots in residential care, in particular regarding robopets, has received much less attention and the need for more research on expectations and preferences in this area has been highlighted (Kachouie et al., 2014).

To improve understanding of the role and effects of robopets for older people in residential care, we conducted a systematic review of the existing qualitative and quantitative research to address the following research questions: (a) What are the experiences, views and perceptions of residents, families/carers and care home staff of interacting with robopets in the older adult residential care setting?, and (b) what are the measured effects of robopets on the health and well‐being of older people living in residential care and of the staff that care for them?

## 2 METHODS

Our review used best practice methods of evidence synthesis (Higgins & Green, 2011) and was developed in consultation with three relevant professionals (care home owner and manager, and a veterinarian) who formed our Expert Advisory Group (EAG). The protocol for the review was registered with PROSPERO (CRD42017081794). The review is reported according to the Enhancing Transparency in Reporting the Synthesis of Qualitative Research (ENTREQ) guidelines and the Preferred Reporting Items for Systematic Reviews and Meta‐Analyses guidelines (Liberati et al., 2009; Tong, Flemming, McInnes, Oliver, & Craig, 2012; see Table S1 and S2).

### 2.1 Literature search

The search strategy was developed by an information specialist (AB) in consultation with experts and used a combination of relevant controlled vocabulary terms (e.g., MeSH) and free text terms. The MEDLINE search strategy is shown in Figure S1. The following databases were searched from inception to April 2017 and updated in December 2018: MEDLINE, EMBASE, PsycINFO, SPP (via OvidSP), CINAHL, AgeLine (via EBSCOhost), CDSR, CENTRAL, DARE (via Wiley Online, Cochrane Library), ASSIA (ProQuest), Web of Science Core Collection, SCOPUS and ProQuest Dissertations and Thesis Global with no date or language restrictions. Forward and backward citation chasing of each included article was performed.

### 2.2 Study selection and eligibility criteria

Eligible articles reported either (a) the views, experiences and perceptions of interacting with robopets of older people resident in care homes, their families and carers and care home staff, or (b) the effects of robopets on health and well‐being (including depression, agitation, loneliness and stress and quality of life), social interaction, engagement, physical function, behavioural symptoms, medication use and adverse events. Robopets were defined as small animal‐like robots which have the appearance and behavioural characteristics of a companion animal or pet.

Qualitative studies using recognised methods of qualitative data collection (such as interviews, focus groups and observations) and of analysis (such as thematic analysis, grounded theory and Interpretative Phenomenological Analysis) were considered eligible for inclusion, as were randomised controlled trials, randomised crossover trials and cluster randomised trials. Eligibility criteria were applied to all unique titles and abstracts by two researchers (RA, NO or RW) independently. The full texts of articles initially considered as meeting the inclusion criteria were retrieved and the eligibility criteria applied in the same way. Discrepancies at both stages were discussed and resolved with another reviewer (JTC) where necessary.

### 2.3 Quality appraisal and risk of bias

We used the Wallace criteria (Wallace, Croucher, Quilgars, & Baldwin, 2004) and Cochrane Risk of Bias Tool (Higgins et al., 2011) to critically appraise the qualitative and quantitative studies, respectively. Qualitative studies were appraised by two reviewers (RA and NO). Quantitative risk of bias was performed by one reviewer (PMcG) and checked by a second (BW), with discrepancies discussed and resolved with a third (JTC).

### 2.4 Logic model: development and use in the review

**Figure 1:**
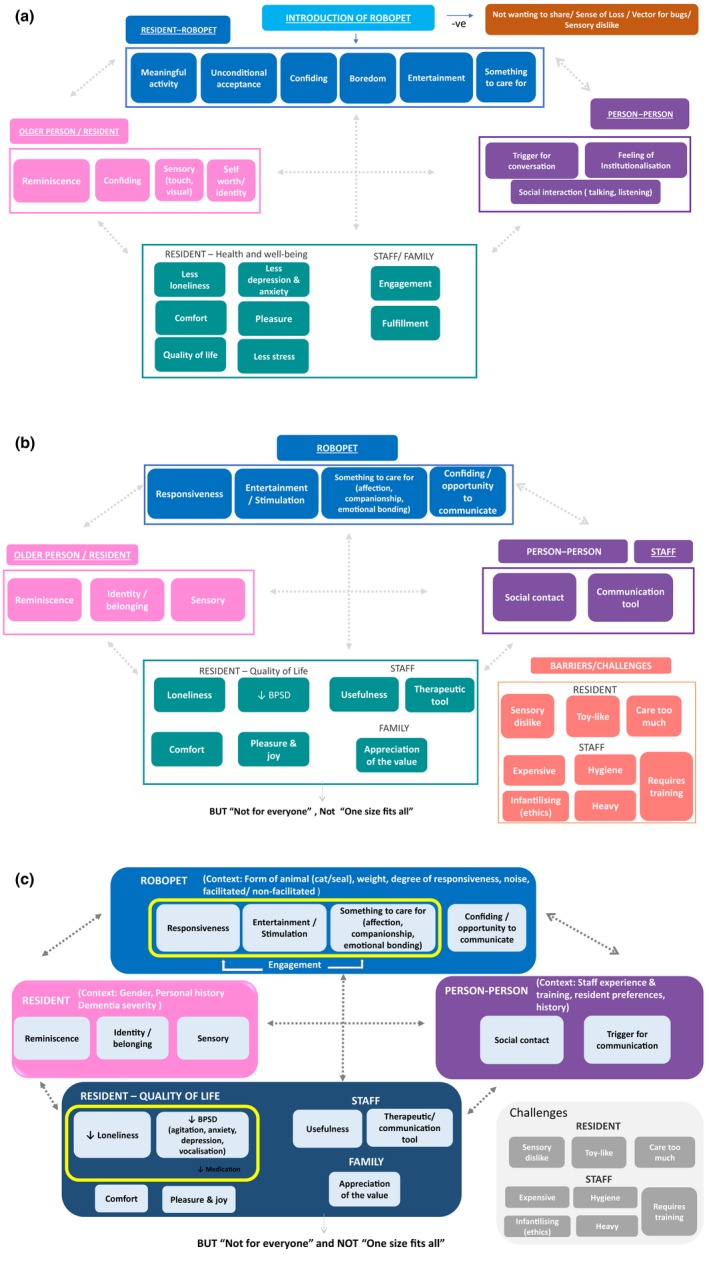
The (a) Initial logic model. (b) Logic model iteration after qualitative data extraction. (c) Final logic model incorporating quantitative findings (highlighted by thick borders) [Colour figure can be viewed at wileyonlinelibrary.com]

At the outset of the review, we developed an a priori logic model (Rohwer et al., 2017) to hypothesise how robopets might influence the health and well‐being of care home residents, staff and family members. The initial logic model was developed by the author team using theoretical literature (Beetz, 2017; Bernabei et al., 2013; Chur‐Hansen, Stern, & Winefield, 2010) and our experience of other reviews in the care home setting (Abbott et al., 2013; Thompson Coon et al., 2014; Whear et al., 2014). We considered the nature of the robopet intervention, the factors that may act as barriers to residents interacting with a robopet, the immediate outcomes (perceived and measured) for the resident and possible mediating factors (see Figure 1a) (Anderson et al., 2011).

The logic model was used as a framework to guide data extraction and synthesis (Booth & Carroll, 2015). Following data extraction and quality appraisal of the qualitative evidence, two reviewers (RA, NO) met to discuss modifications and additions to the model (see Figure 1b). The second iteration was shared with the wider review team and the EAG. We used this second iteration of the logic model as a basis to bring the qualitative and quantitative evidence together. The overarching synthesis resulted in a third and final iteration of the logic model (Figure 1c), to show how the focus and findings from the qualitative and quantitative evidence overlaps and differs.

### 2.5 Data collection

#### 2.5.1 Qualitative studies

The first iteration of the logic model was used as a “scaffolding framework” to extract and code the qualitative data (Booth & Carroll, 2015). The individual elements of the logic model were “deconstituted” to become fields in the data extraction form (Carroll, Booth, Leaviss, & Rick, 2013). Data were extracted on methods, participants, intervention (where relevant) and findings using the initial logic model as a framework of themes against which to code the extracted data. Using a framework in this way is flexible, in that it provides an initial structure but still allows for themes to be iteratively refined, expanded, created or removed, as data from the included paper are collected, coded and synthesised (Rehfuess et al., 2017). Papers with higher methodological quality were extracted first, and the same process was then applied to the papers of lower methodological quality. Two reviewers (RA and NO) independently extracted data from the qualitative papers and met to discuss findings and come to a consensus.

#### 2.5.2 Quantitative studies

Data were collected using standardised, bespoke data extraction forms, piloted for use in this review. Data were extracted by one of three reviewers (RA, RW and PMcG) and fully checked by another (JTC). Data were extracted on the study design, sample characteristics, format and duration of intervention, type of robopet, setting, outcome measures and results. We also collected data on the source of study funding and any conflicts of interest declared by the study authors. Where data were missing, we contacted study authors for further details. Four out of six authors contacted responded to this request.

### 2.6 Data synthesis

#### 2.6.1 Qualitative studies

Two authors who had each read and re‐read the papers discussed whether all components of the model were observed in the data, and whether any new components or underlying themes were evident in the data that were not part of the initial model. The same two authors refined the logic model and a second iteration of the model, which included both modified and new elements that had not been anticipated in the first iteration, was produced and shared with the wider team and EAG for discussion.

#### 2.6.2 Quantitative studies

Random effect meta‐analyses were performed where we had sufficient data from RCTs assessing the same outcome (DerSimonian & Laird, 2015). Pooling was performed on the outcomes measured immediately following the intervention. As we used a random‐effects model for the meta‐analyses, the weightings for each study were determined not only by the size of each study included, but also by between‐study heterogeneity. Unadjusted summary data were used to calculate standardised mean differences (SMDs). As all the outcomes were continuous, pooled effects are reported as standardised mean differences with 95% confidence intervals. Where there were differences in the number of individuals contributing to baseline and follow‐up summary statistics, we used the average sample size.

#### 2.6.3 Overarching synthesis

Combining the qualitative evidence synthesis with the quantitative was performed through a process of mapping findings to the logic model and ongoing discussion amongst the author team (Richardson et al., 2015).

## 3 RESULTS

**Figure 2:**
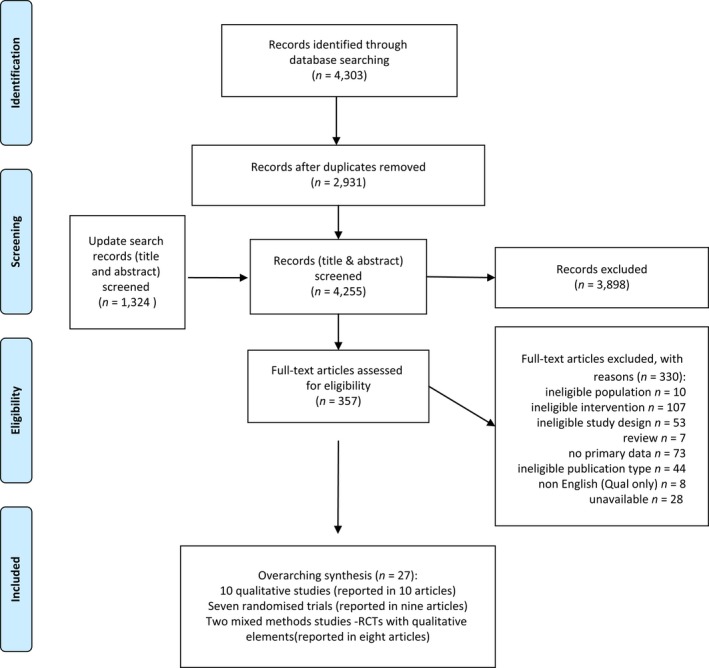
PRISMA flow diagram showing inclusion of articles [Colour figure can be viewed at wileyonlinelibrary.com]

The initial searches identified 2,931 unique papers. Of these, 344 were selected for full‐text review and 19 studies (reported in 27 papers) met the inclusion criteria (see Figure 2 for reasons for exclusion): 10 qualitative studies (Birks, Bodak, Barlas, Harwood, & Pether, 2016; Chang & Sabanovic, 2015; Chang, Sabanovic, & Huber, 2013; Giusti & Marti, 2006; Gustafsson, Svanberg, & Müllersdorf, 2016; Iacono & Marti, 2016; Jung, van der Leij, & Kelders, 2017; Moyle et al., 2016; Niemelä, Määttä, & Ylikauppila, 2016; Pfadenhauer & Dukat, 2015), 2 mixed methods (randomised trials with qualitative elements), reported across 8 papers (Mervin et al., 2018; Moyle, 2017b; Moyle, 2017a; Moyle, 2018a; Moyle, 2018b; Moyle, 2019; Robinson, Macdonald, Kerse, & Broadbent, 2013a, 2013b) and seven randomised trials reported in nine papers (Banks, Willoughby, & Banks, 2008; Joranson, Pedersen, Rokstad, & Ihlebaek, 2015, 2016; Libin & Cohen‐Mansfield, 2004; Moyle et al., 2013; Petersen, Houston, Qin, Tague, & Studley, 2017; Thodberg, Sorensen, Christensen, et al., 2016; Thodberg, Sørensen, Videbech, et al., 2016; Valenti Soler et al., 2015). An update search, carried out in July 2018 across all databases with de‐duping against those already screened, found no additional included papers or studies.

### 3.1 Study characteristics

There were five types of robopet across the 19 studies: 15 studies, in 23 papers, reported on the impact of the robotic seal Paro, one on the robotic cat JustoCat (Gustafsson et al., 2016), one on the robotic cat NeCoRo (Libin & Cohen‐Mansfield, 2004), one on the robotic dog Aibo (Banks et al., 2008) and one on a robotic teddy bear CuDDler (Moyle et al., 2016). CuDDler provoked some discussion amongst the review team in terms of whether it could be considered an animal or pet‐like robot, but reviewers considered the teddy bear to be little different to a seal in terms of whether it constituted being a companion animal, especially as it was given the ability to be “purr when patted, blink its eyes, move its head and invite a hug” (Moyle et al., 2016). Key characteristics of the studies are shown in Tables 1 and 2. Most studies involved assessing the effects or perceived impacts or experiences of specific sessions spent with a robopet. These sessions could be facilitated by therapists or researchers (Birks et al., 2016; Chang et al., 2013; Giusti & Marti, 2006; Gustafsson et al., 2016; Iacono & Marti, 2016; Moyle et al., 2016; Robinson, Broadbent, & MacDonald, 2016) or resident‐led with residents interacting with the robot as they wished. The robots were used in either a group or an individual context, or both. The purpose and content of the therapy sessions could involve diversional/recreational therapy, sensory therapy, narrative therapy or in some cases, left open for the residents to engage as and when they wished. Contact sessions mostly varied from 10‐ to 40‐min sessions per day and from two to three sessions per week, with duration ranging from 4 weeks to approximately 4 months. Outliers to this were one pilot crossover study which evaluated only one 10‐min session with a robopet (Libin & Cohen‐Mansfield, 2004), and two studies which reflected back on experience of a robopet over the duration of 1 year (Jung et al., 2017; Niemelä et al., 2016). One study did not involve specific sessions, but assessed the effect of introducing the robopet into general areas in the care home (Chang & Sabanovic, 2015). Two of the qualitative studies reported on care workers’ experiences of using robopets in care homes not related to a specific intervention study (Jung et al., 2017; Niemelä et al., 2016).

**Table 1:** Study characteristics (qualitative studies)

Study ID	Country	Study description	Participants	Setting (nursing home/care home)	Study aim and context of robopet exposure	Data collection	Analysis (tools used)
Birks 2016	Australia	Qualitative exploratory study	3 recreational therapists	125‐bed aged care facility	To explore the experience of therapists using PARO (baby seal) as a therapeutic tool with a diverse group of residents. Paro was employed daily as a diversional therapy with selected residents in either an individual or group activity. Each session was 30/40 min. Residents encouraged to interactively engage with Paro including stroking, cuddling and speaking. This occurred over 4 months	Semi‐structured interviews for 1 hr each. Each therapist also asked to maintain a journal	Inductive thematic analysis from the verbatim interview scripts and therapists journals
Chang 2013	USA	Observational study	10 residents displaying mild to severe cognitive impairment, 2 therapists	1 nursing home	To explore how using Paro as a multimodal sensory stimulus in a group activity affected the residents’ interactions with Paro itself, other people, the environment, and how the group used Paro; and to understand therapists’ perceptions and reflections on Paro's use in therapy. Paro was introduced to a small group of 4–7 residents as an activity for 8 weeks. The therapist led the activity, showing Paro to the residents and encouraging them to interact with Paro. The activity was open and flexible and residents did not attend all therapy sessions	Interviews before and after with 2 therapists	No details given
Chang 2015	USA	Observational study	Residents with dementia, staff and visitors	1 nursing home	To understand the social shaping process of the PARO seal human–robot interaction Paro was brought into the nursing home 2–3 times a week, for 1 hr per visit. 35 visits were made over a period of 13 weeks. PARO was placed at the centre of the table in specific areas. People were free to interact with PARO as they liked without guidance from the researchers	Informal interviews Observations Semi‐structured interviews with 8 staff	Field notes and interviews coded. Anthropological approach taken for the qualitative analysis to identify patterns
Giusti 2006	Italy	Ethnography	Residents with dementia	1 nursing home	To understand the interpretative dynamics in human–robot interaction by observing nursing home residents interacting with Paro. Paro was introduced in a group activity with 5 residents from 2 different areas of the nursing home twice weekly for 4 weeks. Paro was placed on the table, and the therapist left the residents alone to interact for 20 min. The activity was only partially structured, and residents were free to leave the session at any time	Video recording of 4 activity sessions with Paro	Qualitative speech behavioural analysis
Gustaffson 2015	Sweden	Qualitative interview study	11 professional caregivers (RNs, ANs, OTs)	1 dementia care home	To explore professional caregivers’ experiences of an interactive robotic cat (JustoCat) regarding usability, function and effects. Cat introduced as an activity for 7 weeks. Staff were trained by an OT in how to use JustoCat and encouraged staff to talk about JustoCat with the residents and were shown how to stroke it and make it purr. Early sessions were supervised, and the importance of staying with cat was emphasised	Qualitative interviews (used an interview guide to collect narratives relating to the impact/meaning and use of JustoCat in daily/working life, and its functionality)	Analysed using a qualitative descriptive approach, in which patterns were formulated into categories to present the variations in experiences
Iacono 2016	Italy	Narrative therapy study	6 residents with dementia	1 care home	To explore the potential of Paro as a tool for counteracting cognitive deterioration in residents with dementia through non‐pharmacological treatment based on narrative activity. Paro and Sugar (stuffed toy which looks like a seal pup) were introduced to 2 groups of residents (three residents in each group). Each group had 6 sessions with Paro and 6 with Sugar. 3 weekly sessions of 30 min each were conducted over 4 weeks (a total of 12 sessions). The goal of each session was for residents to create stories collaboratively	Video recording of sessions and writing of stories at end of sessions	Narrative analysis–calculate the number of words in each individual story; analyse the story on the basis of the model used in its construction—characters, setting and plot; analyse the settings, how they were described and the plot (which is the story itself and how it evolves over the course of the narration)
Jung 2017	Netherlands	Mixed methods	5 healthcare providers with experience of using Paro	1 care facility	To focus on how people with dementia could benefit from interaction with an animal‐like robot companion that is able to understand and respond to different types of touch. Paro had been available to the healthcare providers for 1 year. Their amount of experience with Paro differed because they cared for different patients with different needs	Semi‐structured interviews	Inductive approach
Moyle 2016	Australia	Pilot feasibility study	5 residents with dementia	107‐bed nursing home	To determine how feasible, effective and tolerable the CUDDLER bear robot was for people with dementia in a nursing home setting. Participants were offered 3 x 30 min sessions per week, for 5 weeks, with CUDDLER in a quiet closed room. Each session there was a facilitator (Registered Nurse with dementia care experience) whose aim was to encourage interaction with CUDDLER using a series of questions	Semi‐structured interviews (series of six questions), conducted by the intervention facilitator	Audio data from interviews/questions analysed with thematic analysis, concentrating on feasibility, tolerability, effectiveness and reliability
Moyle 2019^a^	Australia	Qualitative	5 participants from a cluster RCT	28 LTC facilities were enrolled in the RCT in South East Queensland	To provide critical reflection and commentary on the potential heterogeneity in responses to Paro during a 10‐week trial. Paro was given to the participant by the Research Assistant and left to interact with it as they wished, wherever they happened to be at the time, unless in the bathroom	Video recordings of participants for 30 min, immediately before (15 min) and during (15 min) sessions at weeks 1, 5 and 10	Coded in Noldus Observer XT using the Video Coding Incorporating Observed Emotion Scheme (Jones, Sung & Moyle, 2015)
Moyle 2017a^a^	Australia	Qualitative study nested within a larger RCT	20 family members, 10 from Paro and 10 from Plush Toy study conditions	9 LTC facilities	To explore the perceptions of family members about the use of Paro and Plush Toy with their older relative with dementia in LTC. Paro was compared with a look‐alike Plush Toy and usual care. Participants received individual, non‐facilitated, 15‐min sessions three afternoons per week (Monday, Wednesday and Friday between 1.00 p.m. and 5.00 p.m.) for 10 weeks with either Paro or a Plush Toy	Semi‐structured interviews (seven areas of questioning)	Inductive thematic analysis (Braun & Clarke, 2006)
Moyle 2018a^a^	Australia	Qualitative interview study as part of Cluster RCT	20 staff (Facility manager, clinical nurse consultants, RNs, EENs, Activity coordinators, NAs)	Nine long‐term care facilities across SE Queensland	To explore care staff perceptions of PARO baby seal (version 9) versus a comparative look‐alike non‐robotic animal as regards to the benefits and limitations in dementia care Participants received 3 x 15 min non‐facilitated sessions per week, for 10 weeks, with PARO. A trained RA left the resident with PARO using a prescribed script. Residents could interact with PARO as they liked. The care home staff were not involved in facilitating these sessions, but had opportunities to observe the sessions	Semi‐structured interviews that focussed on seven main areas, additional specific questioning was possible in response to interview responses.	Inductive thematic analyses were used to converge and compare themes. Themes were clustered according to views and experiences and linked to primary outcomes of interest
Niemela 2016 (PC)	Finland	Qualitative interviews study	1 Director Nurse (decision‐maker for municipality nursing homes) 10 professional carers (nurses)	3 Nursing Homes	To explore the process and criteria of adopting robots in elderly care in Finland and experiences of their use All three nursing homes had access to Paro for at least 1 year. Paro circulated between departments so the caregivers in one department typically used Paro 1–2 months at a time and could have periods of several months of non‐use	Semi‐structured interview for Director Nurse and Focus Group interviews for nurses.	NA
Pfadenhauer 2015	Germany	Ethnography		1 residential care centre for the elderly (with dementia)	To explore how people incorporate social robotics into their social interactions, and thereby changing these interactions. Paro was used by two care workers; three sessions per month, all but one session was in a group session	Participant observations and videographic documentation	“Quasi‐Socratic interpretation technique”—researcher gives his peers an exhaustive account of his/her (ad‐hoc) interpretation of a text or a video sequence. Ensuing discourse prompts reflection and revision of interpretation
Robinson 2013^a^	New Zealand	Qualitative interview study with intervention arm of an RCT	16 residents with dementia and 21 staff (manager, activity coordinator, nurses and caregivers)	1 retirement home with both hospital and home areas	To understand how residents and staff interact with PARO (baby seal) especially within the group format. Two sessions a week over 12 weeks: semi‐structured and resident directed. Topics for discussion available if residents not engaged in their own conversations. Passing round of PARO encouraged	Interviews with residents. Written questions for staff Observation notes during group sessions	Codes from previous robot—human research used and added to. Authors reported “data analysed and common themes noted.”

a Qualitative study encased within RCT (see Table 2 for RCT detail).

**Table 2:** Study characteristics (randomised trials and mixed methods studies)

Study ID and date	Country	Study design	Sample	Setting (nursing home/ care home)	Intervention arm description	Comparator arm(s) description	Outcomes measured (tools used)
Banks 2008	USA	3 arm RCT	*N* = 42. Residents, Mixed‐sex, Mean age: NA	Nursing home	Individual sessions with AIBO robotic dog: 30 min for 8 weeks AIBO kept stationary in cradle Note: not clear whether staff facilitated	Living dog or routine care (no AAT). Intervention with dog as described for AIBO	Loneliness (University of California Los Angeles (UCLA) loneliness scale) Attachment (modified Lexington Attachment to Pets Scale [LAPS])
Joranson 2015	Norway	Cluster RCT	*N* = 60. Residents with dementia, Mixed‐sex, Mean age: INT (83.9 years), COMP (84.1 years)	Nursing home	Group sessions with PARO robotic seal: 30‐min sessions, twice a week, over 12 weeks Staff facilitated	Routine care, details not described	Agitation (validated Norwegian version of Brief Agitation Rating Scale (BARS)) Depression (validated Norwegian version of Cornell scale for Symptoms of Depression in Dementia (CSDD)) Medication (overviews of medication in accordance with Anatomical Therapeutic Chemical ATC Classification System)
Joranson 2016	Norway	Cluster RCT	As above	As above	As above	AS above	Agitation (validated Norwegian version of Brief Agitation Rating Scale [BARS]) QoL (The Quality of Life in Late‐Stage Dementia (QUALID) scale) Medication usage (overviews of medication in accordance with Anatomical Therapeutic Chemical ATC Classification System)
Libin and Mansfied 2004	USA	Pilot RCT (crossover)	*N* = 9 Residents with dementia All female Mean age 90 years	Nursing home	Individual session with NeCoRo robotic cat: 10 min Staff facilitated	Individual session with a plush cat 10 min Staff facilitated	Agitation (Agitated Behaviors Mapping Instrument) Affect (Lawton's Modified Behavior Stream) Engagement (Bespoke observation tool)
Moyle 2013	Australia	RCT (crossover)	*N* = 18 Residents with dementia Sex not reported Mean age 85.3 years	Both (residential care with 62 nursing home beds)	Group sessions with PARO 45 min, 3 times a week, over 5 weeks Staff facilitated Note: 2 different PAROs used to allow participants more individual time with robot	Reading control group Staff directed Activities include being read to, looking at pictures and social interaction in group through engaging in questions about reading Delivered as in PARO group	QoL (Quality of Life in Alzheimer's Disease Scale (QOL‐AD)) Anxiety (Rating Anxiety in Dementia Scale (RAID)) Apathy (Apathy Evaluation Scale (AES)) Depression (Geriatric Depression Scale (GDS)) Wandering (Revised Algase Wandering Scale‐Nursing Home version (AWS)) Mood state (Observed Emotion Rating Scale (OERS))
Moyle 2017b	Australia	3 arm Cluster RCT	*N* = 415 Residents with dementia, Mixed‐sex, Mean age: INT (84 years), COMP routine (86 years), COMP toy (85 years)	Nursing home	Individual sessions with PARO 15 min, 3 times a week, for 10 weeks Individual left to interact	Routine care (not described) Plush toy (PARO without batteries) group receives same contact sessions as PARO group	Engagement (30 min Video observation by trained RAs) Mood states (30 min Video observation by trained RAs) Agitation (30 min Video observation by trained RAs plus Cohen‐Mansfield Agitation Inventory Short Form (CMAI‐SF))
Moyle 2018b	As above	As above	As above	As above	As above	As above	Sleep and day and night time activity (Sensewear armband)
Mervin 2017	As above	As above	As above	As above	As above	As above	Cost‐effectiveness
Petersen 2017	USA	RCT	*N* = 61 Residents with dementia, Mixed‐sex Mean age: INT (83.5yrs), COMP (83.3 years)	Unclear—could be assisted or independent living	Group sessions with PARO 20 min, 3 times a week, for 12 weeks Staff facilitated	Routine care – physical activity, music, mental stimulation activities Staff facilitated small groups Daily	Rating for anxiety in dementia (RAID) Depression in dementia (Cornell Scale for Dep in Dem) Severity of dementia (Global Deterioration Scale) Pulse rate (stress/anxiety), Pulse oximetry (stress/anxiety) Galvanic skin response (arousal) Medication (medical records)
Robinson 2013	New Zealand	RCT	*N* = 40 Residents with and without dementia, Mixed‐sex Age range: 55−100yrs	Both (retirement home with both hospital and home areas)	Group sessions with PARO Duration not stated, 2 times a week, for 12 weeks Staff facilitated groups Some individual sessions if participant unavailable for the group	Routine care included bus trips during time of intervention group sessions or an alternative activity (crafts, movie, bingo) also included the presence of a live dog at times	QoL (Quality of Life for Alzheimer's Disease (QoL‐AD) Depression (Geriatric Depression Scale (GDS) Loneliness (UCLA Loneliness scale) Interaction (observations)
Thodberg 2016	Denmark	3 arm RCT block design	*N* = 100 Residents with and without dementia, Sex and age reported across nursing home sites	Nursing home	Individual sessions with PARO 10 min, 2 times a week, for 6 weeks Individual left to interact	Living dog Project staff facilitated sessions with a visiting living dog Toy cat Staff facilitated sessions with soft toy cat which was not interactive Both group sessions delivered as in PARO group	Cognitive state (mini‐mental state examination MMSE) Disability (Gottfries‐Brane‐Steen scale (GBS)) Depression (Geriatric depression scale (GDS)) Sleep (accelerometers based on actigraphy technology) BMI
Thodberg 2016	Denmark	As above	As above	As above	As above	As above	Physical contact with visiting animal (direct observation) Talk directed to animal and visiting person (direct observation) Visual contact with either animal or visiting person (direct observation) Cognitive state (MMSE) Disability (GBS) Depression (GDS)
Valenti Soler 2015	Spain	3 arm Cluster RCT	*N* = 110 Residents with dementia, Mixed‐sex, Mean age 84.7yrs across all groups	Nursing home	Group sessions with PARO 30–40 min, twice a week, for 12 weeks staff facilitated	Routine care—no further description Living dog Staff facilitated sessions with living dog Group based as described for PARO group	Dementia deterioration (Global Deterioration Scale) Dementia severity (MMSE) Apathy (apathy scale for institutionalised patients with dementia in nursing home environments) Neuropsychiatric inventory QoL (QUALID)

Abbreviation(s): COMP, Comparator group; INT, Intervention group; RCT, randomised controlled trial.

All studies were conducted within the past 15 years. Five studies were conducted in the United States (Banks et al., 2008; Birks et al., 2016; Chang & Sabanovic, 2015; Chang et al., 2013; Petersen et al., 2017) and four in Australia (Birks et al., 2016; Moyle et al., 2013, 2016, 2017a), with the remaining studies taking place in Italy (Giusti & Marti, 2006; Iacono & Marti, 2016), New Zealand (Robinson, Macdonald, Kerse, & Broadbent, 2013a), Denmark (Thodberg, Sorensen, Christensen, et al., 2016), Finland (Niemelä et al., 2016), Germany (Pfadenhauer & Dukat, 2015), the Netherlands (Jung et al., 2017), Norway (Joranson, Pedersen, Rokstad, & Ihlebaek, 2015), Spain (Valenti Soler et al., 2015) and Sweden (Gustafsson et al., 2016). The studies involved more than 800 residents (the total number is not clear as two observation studies did not report the number of residents observed) (Chang & Sabanovic, 2015; Pfadenhauer & Dukat, 2015). Just over half of the studies had a focus on the use of robopets for residents with dementia. Seventy‐nine members of staff (descriptions varied from therapists, activity coordinators, professional caregivers—nurses, occupational therapists, healthcare providers) and 23 family members were included in the qualitative studies.

### 3.2 Quality of the evidence

#### 3.2.1 Qualitative papers

All but one of the papers (Chang et al., 2013) stated a clear research question, all used appropriate study designs and most adequately described how data were collected. In all of the studies, the sample was assessed as being drawn from the appropriate population, and in all but one (Chang et al., 2013), the reported findings were substantiated by the data shown. Three studies noted a theoretical perspective, and it clearly influenced the study design (Giusti & Marti, 2006; Iacono & Marti, 2016; Moyle et al., 2019). In three of the studies, it was difficult to appraise the data collection and analysis due to inadequate reporting (Chang et al., 2013; Niemelä et al., 2016; Pfadenhauer & Dukat, 2015) (see Table S3).

#### 3.2.2 Quantitative studies

A low risk of bias for random sequence generation was observed for the majority of the trials, suggesting that selection bias across the studies was low. Most studies performed poorly in terms of the blinding of participants and personnel, with only one study at a low risk of bias for this criterion (Moyle et al., 2017b). The majority of the studies performed power calculations, and 4 of the trials clearly accounted for all of their participants in the reporting of the studies (Joranson et al., 2015; Moyle et al., 2013, 2017a; Robinson et al., 2013a). Five trials clearly reported eligibility criteria (Joranson et al., 2015; Moyle et al., 2013, 2017a; Petersen et al., 2017; Valenti Soler et al., 2015). Overall, there was a high proportion of items rated as unclear due to the presence of sizable gaps in reported information for several risk of bias criterions (see Figure S2).

### 3.3 Synthesis

The qualitative evidence synthesis, which guided the overall synthesis, is presented first followed by the evidence on effectiveness from the randomised controlled studies, and finally an overarching synthesis brings the two evidence bases together.

#### 3.3.1 Qualitative synthesis

The qualitative synthesis identified six main components: robopet‐resident engagement, resident, person–person interaction, perceived impact on resident quality of life, staff and family appreciation, and challenges to using robopets. Each component had a set of underlying themes within it, and Table S4 shows which studies contributed to each theme (Table S5 presents additional detail on the themes with illustrative quotations).

### 3.4 Component 1: Robopet‐resident engagement

This consisted of the following themes: responsiveness; entertainment and stimulation; something to care for; and opportunity to communicate and confide.

#### 3.4.1 Responsiveness

Positive behavioural responses were demonstrated through residents touching, petting, stroking, holding and hugging the robopet (Birks et al., 2016; Iacono & Marti, 2016; Jung et al., 2017; Moyle et al., 2019, 2016). Visual responses (Birks et al., 2016; Gustafsson et al., 2016; Moyle et al., 2018a) were often reported in terms of “alertness” and staff involved in a trial perceived that residents appeared to be more alert when they participated in activities with Paro (Moyle et al., 2018a). One professional caregiver reported on an intervention with JustoCat in a care home and highlighted how she perceived an “introverted” resident to have had moments of “‘waking up” and becoming “more aware and alert” (Gustafsson et al., 2016). Interacting with robopets induced verbal responses with residents talking to the robopets either directly or with others (Birks et al., 2016; Chang & Sabanovic, 2015; Giusti & Marti, 2006; Iacono & Marti, 2016; Moyle et al., 2016; Robinson et al., 2016). Verbal responses were often positive showing appreciation for the robot using words such as “beautiful” and “cute” (Giusti & Marti, 2006; Iacono & Marti, 2016; Moyle et al., 2016; Robinson et al., 2016).

However, some studies offered descriptions of residents being uninterested in responding to the robopet (Birks et al., 2016; Moyle et al., 2019, 2016). There were residents who refused to interact with Paro to any significant degree and only did so when asked, and there was one example where the presentation of Paro evoked a strong verbal and behavioural negative response (Moyle et al., 2019). Residents’ responses were observed to change over time; for some residents, their responses changed from negative to positive and could even vary day‐to‐day, and for others, responsiveness decreased over time as the robopet blended into their everyday routines (Birks et al., 2016; Chang & Sabanovic, 2015; Moyle et al., 2019, 2016). Staff perceived that residents’ responses could vary according to whether they were living with dementia and according to the severity of the dementia (Birks et al., 2016; Chang & Sabanovic, 2015; Jung et al., 2017).

#### 3.4.2 Entertainment and stimulation

Robopets were described as a way of entertaining and diverting residents who were “restless or sad” (Jung et al., 2017) and “bored” (Moyle et al., 2017b). They could provide an opportunity for “humour and play” (Gustafsson et al., 2016). The robopets also acted as a means of stimulating residents’ curiosity which was demonstrated in “talking to” the robopet and in “talking with others” about the robopet (Chang & Sabanovic, 2015; Giusti & Marti, 2006). However, not all residents found robopets stimulating and entertaining and reported feeling bored (Robinson et al., 2016).

#### 3.4.3 Something to care for

Residents were observed treating the robopets as they would real pets, displaying affection (e.g., hugging, petting, kissing and stroking; Robinson et al., 2016). Verbal responses from residents also indicated that they regarded them as live creatures (Chang & Sabanovic, 2015; Giusti & Marti, 2006; Iacono & Marti, 2016):

“S5: …when you will be grown up, I will take a…what [sic] the name of that thing (mimicking a leash)

Other woman: leash!

S5: …I will take a leash and I will put it around your neck (Paro moves its head) no? (talking to others about Paro…) Look at him he understand [sic] everything!”(Giusti & Marti, 2006)

However, there were those residents who could develop an “emotional attachment” to the robopet fully aware that it was not “real”: “I know it is an inanimate object but I can't help but love her*”* (Robinson et al., 2016).

#### 3.4.4 Opportunity to communicate and confide

Robopets provided residents with an opportunity to communicate and confide their innermost thoughts, feelings and even frustrations (Birks et al., 2016; Chang & Sabanovic, 2015; Robinson et al., 2016): “I woke up today and thought, today is going to be a good day because I get to see my friend.” (Robinson et al., 2016). Observations of residents’ interactions with Paro showed that it could act as a “conversational partner” (Chang & Sabanovic, 2015), with residents’ conversations ranging from everyday matters in the “here and now” to the more personal and emotional: “…Did they go off and leave you here? My son left me here, I want to go home but I can't’” (Chang & Sabanovic, 2015).

### 3.5 Component 2: Resident response

This encompassed three themes which could impact on the degree to which the resident responded to the robopet: reminiscence; sensory experience; and identity/belonging.

#### 3.5.1 Reminiscence

Five studies noted that robopets appeared to awaken memories which increased communication with care staff and family members (Birks et al., 2016; Gustafsson et al., 2016; Moyle et al., 2017b, 2016; Pfadenhauer & Dukat, 2015). In some cases, the robopet could facilitate more focussed memories of specific activities or time spent with animals and pets: “…Participant “J” could not visualise CuDDler …the texture and fur reminded “J” of her recently deceased dog…CuDDler evoked fond memories of the animal she missed holding and touching” (Moyle et al., 2016).

#### 3.5.2 Sensory experience

Six studies reported on the aesthetic appeal of the robopets and how they engaged the residents’ visual, tactile and auditory senses (Birks et al., 2016; Giusti & Marti, 2006; Gustafsson et al., 2016; Iacono & Marti, 2016; Jung et al., 2017; Robinson et al., 2016). Paro, in particular, was seen as attractive and residents enjoyed touching, stroking and holding it: “...when they [patients] hold him [Paro] he lifts his head and as a result the whiskers move along their faces which is a very sensitive area for these people, they can feel it clearly” (Jung et al., 2017). The weight and size of the robopet also impacted on the senses: professional caregivers judged JustoCat to have “natural size and weight” (Gustafsson et al., 2016), offering a sense of stability and comfort to residents. However, some residents expressed their dislike of the robopets in sensory terms, and both Paro and CuDDler were described by residents as “too heavy” and “too mechanical” (Moyle et al., 2016; Robinson et al., 2016). Whilst JustoCat's response to stroking by purring was praised by staff, other studies noted staff descriptions of Paro's auditory responses as “…repetitive, irritating, too loud and too high pitched” (Jung et al., 2017).

#### 3.5.3 Identity/Belonging

The “individual history” (Chang & Sabanovic, 2015) or “biography” (Moyle et al., 2019) of residents could influence how they responded to the robopets. One study suggested that like or dislike of animals could be an important factor (Moyle et al., 2019), and another study found that gender affected verbal and behavioural responses to Paro; for example, women showed appreciation of Paro's appearance and movement and many talked to Paro as if it were alive, whereas the men responded to Paro as a toy or tool and appreciated its technical functions (Chang & Sabanovic, 2015).

There was a belief by staff that Paro provided a sense of belonging for residents and replaced family as it “…takes them back into a space in their life where they feel loved” and “…gives them a sense of belonging and warmness, and builds up their confidence”(Moyle et al., 2018a) Positive resident responses to Paro enhanced the care home environment and were perceived by staff as being important in “building a community*”* (Moyle et al., 2018a).

### 3.6 Component 3: Person‐to‐person interaction

This captures the social aspect of the person‐to‐person interaction whereby the robopet triggered conversation and enhanced social contact between residents and with staff and family.

#### 3.6.1 Social contact

The social aspects of robopets were highlighted by many studies (Birks et al., 2016; Chang & Sabanovic, 2015; Chang et al., 2013; Giusti & Marti, 2006; Gustafsson et al., 2016; Jung et al., 2017; Moyle et al., 2018a, 2017b, 2016; Pfadenhauer & Dukat, 2015; Robinson et al., 2016). A robopet could act, not only as a “conversational partner” (Chang & Sabanovic, 2015) for individual residents, but also as a conduit for communicating with others. Residents were observed “talking to” and “talking about” the robopets, and staff perceived that “talking to” the robopet gave residents confidence to talk to others (Moyle et al., 2018a). The robopets also served as an “icebreaker” between staff and residents, and staff were overheard “joking and laughing” with residents about the robopet (Robinson et al., 2016). Robopets were reported to enhance social interactions between residents and family members, particularly in the later stages of dementia. A therapist observed how Paro facilitated an “expression of affection” (p. 3) between a resident who could not speak and her husband: “…you could see the look on her face and his face and the touching which would—she touched his hand and they both touched Paro” (Birks et al., 2016). Family members also suggested that robopets helped in day‐to‐day conversation and provided a diversion from the usual topics of conversation (Gustafsson et al., 2016; Moyle et al., 2017b).

### 3.7 Component 4: Resident quality of life

This relates to the perceived benefits impacting resident quality of life and consisted of four themes: reduced loneliness; increased pleasure and joy, increased comfort and safety; and reduced behavioural and psychological symptoms of dementia (BPSD).

#### 3.7.1 Reduced loneliness

Staff and family believed that holding, touching and talking with the robopet reduced the loneliness experienced by some residents (Birks et al., 2016; Gustafsson et al., 2016; Jung et al., 2017; Moyle et al., 2018a, 2017a, 2016; Robinson et al., 2016), and this was particularly relevant for those who spent more time by themselves, or who did not routinely engage in the usual activities of the care home: “Just to calm the residents down, or the residents who are very lonely and they don't participate in any activities” (Moyle et al., 2018a). The residents also commented on how their time with the robopet made them feel less alone. (Gustafsson et al., 2016; Robinson et al., 2016).

#### 3.7.2 Increased pleasure and joy

Resident feedback and observations from both staff and family members showed that engaging with the robopets increased pleasure and joy for residents (Birks et al., 2016; Chang & Sabanovic, 2015; Gustafsson et al., 2016; Moyle et al., 2018a, 2017b, 2016; Robinson et al., 2016): “Mum just loved it. She talked to it. She had a smile on her face as wide as the Great Australian Bite. It definitely made a difference to her mood” (Moyle et al., 2017b). It did not appear to matter whether the robopet was perceived as artificial or real: “It doesn't matter, because I can see that the robotic cat has an impact on my dad's quality of life” (Gustafsson et al., 2016).

#### 3.7.3 Increased comfort and safety

Staff believed that the robopets brought comfort to the residents and described the “soothing” and “calming” influence of Paro and JustoCat, particularly when residents were anxious or upset (Birks et al., 2016; Chang & Sabanovic, 2015; Chang et al., 2013; Gustafsson et al., 2016; Moyle et al., 2018a; Robinson et al., 2016): “…some staff, such as the nurses, started borrowing Paro to comfort anxious dementia residents” (Chang & Sabanovic, 2015). A therapist observed that Paro brought comfort to residents at the end of life by helping them to verbalise their feelings: “I used it on a palliative care resident…she was able to verbalise how she was feeling…she could see that she was thinking about her thoughts and she wanted to pass it on to somebody” (Birks et al., 2016).

#### 3.7.4 Reduced symptoms of BPSD (including agitation, anxiety, depression, vocalisation and associated medication use)

Reductions in anxiety, agitation and vocalisation were frequently noted by staff and family (Birks et al., 2016; Chang et al., 2013; Gustafsson et al., 2016; Jung et al., 2017; Moyle et al., 2018a, 2017b, 2016): “[the resident] sings all the time and it's repetitive and it's very loud. When she has the seal, it stops” (Moyle et al., 2018a). There was a suggestion that using robopets could reduce restlessness and wandering (Moyle et al., 2018a), and in one case, the robopet was used as a complement to/replacement for sedative medication (Gustafsson et al., 2016).

However, some staff thought that the Paro's vocal sounds could overstimulate residents and “elevate rather than diminish agitation” (Moyle et al., 2018a) and commented on an example of a resident who, when handed Paro, “[g]ot quite aggressive so it didn't seem to help her at all” (Moyle et al., 2018a). Reflections on a cluster randomised control trial on Paro led the authors to conclude that trying to involve uninterested residents with a robopet can increase agitation (Moyle et al., 2019).

### 3.8 Component 5: Staff and family appreciation

The positive reactions of staff towards robopets were mentioned in a number of studies (Birks et al., 2016; Chang & Sabanovic, 2015; Gustafsson et al., 2016; Jung et al., 2017; Moyle et al., 2018a; Niemelä et al., 2016; Robinson et al., 2016), and many staff referred to it as a “tool” for communication, stimulation and entertainment; part of a “therapeutic toolbox” (Birks et al., 2016) to draw on when working with residents and with those with dementia. Paro was described as “very convenient” and a “wonderful support” (Moyle et al., 2018a) when residents were agitated and challenging and helped staff give “good care” (Niemelä et al., 2016) to the residents. One study recorded how the therapists believed that using Paro had enriched their personal lives and deepened their relationship with individual residents (Birks et al., 2016). Staff could be positive about robopets as they considered the alternatives to have greater limitations: “…the seal is clean, it doesn't need feeding, doesn't soil the carpet and the floor” (Moyle et al., 2018a).

Negative staff reactions were also reported (Birks et al., 2016; Niemelä et al., 2016) with Paro described as a “waste of money”(Birks et al., 2016) but other studies also recorded how staff opinions changed positively over time—after observing the residents interacting with the robopet—and changes were made to staff daily routines as they found other ways to use Paro in their care work (Chang & Sabanovic, 2015; Robinson et al., 2016).

Family members appreciated the therapeutic benefits provided by the robopets and how they enhanced the residents’ quality of life (Birks et al., 2016; Gustafsson et al., 2016; Moyle et al., 2017b) and made their visits easier: “if I have my dog or there is the seal, she concentrates on that rather than repetition…It certainly makes the visit easier…” (Moyle et al., 2017b). However, the issue of Paro being toy‐like could lead to disquiet (Moyle et al., 2017b) or stronger negative reactions from some family members (Birks et al., 2016).

### 3.9 Component 6: Challenges to using robopets

Residents articulated their dislike by highlighting specific sensory and toy‐like features of the robopets (Moyle et al., 2016; Robinson et al., 2016). Residents could also display excessive attachment to the robopets with detrimental effects for the individual and for relationships with other residents (Gustafsson et al., 2016; Moyle et al., 2019). Staff were aware that sharing the robopets could be an issue and although there was a suggestion of “[h]aving more to each individual” (Robinson et al., 2016), this was not considered feasible as the cost of Paro (Birks et al., 2016; Jung et al., 2017; Moyle et al., 2018a, 2019; Niemelä et al., 2016) made it unlikely that some care homes could afford to have one at all. Staff were concerned that using robopets may have evoked feelings of infantilisation for residents (Moyle et al., 2018a) and in some cases led to negative reactions from staff, with Paro being dismissed as “that toy” (Birks et al., 2016). Care staff themselves recognised that they should understand how residents react to robopets and in which situations it was appropriate to use the robopet: “You'd have to have the staff who understood exactly how to use them and when to use them and who to use them with” (Moyle et al., 2018a). That training should encompass maintenance of the robopet, including infection control procedures, was also mentioned (Moyle et al., 2019).

Five studies stated that robopets were not for all residents (Birks et al., 2016; Jung et al., 2017; Moyle et al., 2018a, 2019; Robinson et al., 2016) and should not be used as “‘a one size fits all’ approach to care” (Moyle et al., 2018a). Studies noted the diversity of staff opinion as to whom robopets suited (Birks et al., 2016; Jung et al., 2017; Moyle et al., 2018a; Robinson et al., 2016): some questioned its suitability for residents with normal cognitive health (Jung et al., 2017), others queried its value for every resident with dementia (Gustafsson et al., 2016; Jung et al., 2017; Moyle et al., 2018a), and in one case, staff reported concerns that residents with severe dementia may not be able to display whether they want Paro or not (Moyle et al., 2018a).

### 3.10 Quantitative synthesis

Results are presented by outcome (see Table S6).

#### 3.10.1 Loneliness

**Figure 3:**
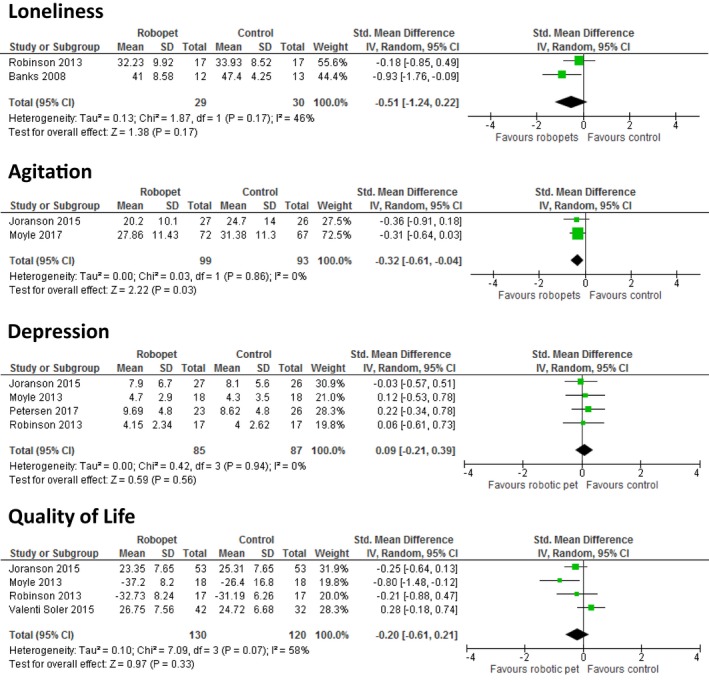
Meta‐analyses showing effect of robopets compared to control activity/usual care on (a) loneliness, (b) agitation, (c) depression and d) quality of life [Colour figure can be viewed at wileyonlinelibrary.com]

Two studies, in a mixed care home population and residents without dementia, respectively, assessed the effect of robopets (one using Paro; Robinson et al., 2013a and one using Aibo; Banks et al., 2008) compared to usual care on loneliness. Whilst both studies reported significant decreases in loneliness for the robopet groups compared to control, the pooled SMD for effect on loneliness did not reach significance (−0.51 [95% CI −1.24 to 0.22, *p* = 0.17], see Figure 3). Of interest, however, the decrease in loneliness in the study by Banks and colleagues (Banks et al., 2008) was comparable to that of the third arm of the study which compared visits by a real dog.

#### 3.10.2 Agitation

Three studies, all involving residents with dementia, assessed the effects of robopets on agitation. Two of the studies (Joranson et al., 2015; Moyle et al., 2017b), comparing Paro to a standard‐care control, provided data enabling pooling: the pooled SMD for effect on agitation was −0.32 (95%CI −0.61 to −0.04, *p* = 0.03), see Figure 3. The third study, a pilot study comparing a robotic cat to a plush toy in a crossover trial, reported no significant effect on agitation (Libin & Cohen‐Mansfield, 2004).

#### 3.10.3 Depression

Five studies, all investigating Paro, reported on the effects on depression: three studies in residents with dementia and two in mixed care home populations (Robinson et al., 2013a; Thodberg, Sorensen, Christensen, et al., 2016). Data from four of the studies were suitable for pooling (Joranson et al., 2015; Moyle et al., 2013; Petersen et al., 2017; Robinson et al., 2013a). The SMD of effect of Paro intervention on depression compared to usual care was 0.09 (95%CI −0.21 to 0.39, *p* = 0.56), see Figure 3. No evidence of effect on depression was also reported in the study by Thodberg, Sørensen, Videbech, et al. (2016) that could not be included in the pooled analysis.

#### 3.10.4 Quality of life

The effect of PARO on quality of life was assessed in three studies with residents with dementia (Joranson et al., 2015; Moyle et al., 2013; Valenti Soler et al., 2015) and one in a mixed care home population (Robinson et al., 2013a). Pooling of data from four studies showed no evidence of overall effect of robopet intervention compared to usual care on quality of life with a pooled SMD of −0.21(95%CI −0.61 to 0.21, *p* = 0.33), see Figure 3.

#### 3.10.5 Engagement/Interaction

Four studies (Libin & Cohen‐Mansfield, 2004; Moyle et al., 2017b; Robinson et al., 2013a; Thodberg, Sørensen, Videbech, et al., 2016), two of which had a focus on residents with dementia, reported on the effects of robopet intervention on engagement and interaction. The data were not suitable for pooling. Three studies reported significantly improved engagement/interaction with Paro compared to control group/normal activities. Paro was found to be more effective in encouraging verbal and physical engagement compared to a plush toy (Moyle et al., 2017b) and was found, alongside a living dog intervention, to initiate the most interaction in terms of physical contact (*p* < 0.001), eye contact (*p* < 0.001) and verbal communication (*p* < 0.05) when compared to a usual care control (Thodberg, Sørensen, Videbech, et al., 2016). Paro was also talked to and stroked significantly more than a resident dog and a greater number of residents engaged in conversation during Paro sessions when compared to sessions with a resident dog and normal activities (*p* < 0.001; Robinson et al., 2013a). A robotic cat did not increase engagement more than a similar looking plush toy in the small crossover pilot study of residents with dementia by Libin and colleagues (Libin & Cohen‐Mansfield, 2004).

#### 3.10.6 Anxiety

Three studies investigated the impact of Paro on anxiety (Moyle et al., 2013, 2017b; Petersen et al., 2017). Pooling of the data was not possible due to missing estimates of data variation. Two of the three studies reported no significant difference in anxiety in Paro groups compared to usual care/control (Moyle et al., 2013, 2017b). Petersen and colleagues (Petersen et al., 2017) reported a significant reduction in the levels of anxiety with the Paro compared to routine care control; however, this different did not take into account the lower levels of anxiety in the control group at baseline.

#### 3.10.7 Medication

Three studies, all involving Paro, investigated the impact of robopets on medication usage. Pooling of the data was not possible. Two studies (Joranson et al., 2015; Mervin et al., 2018) found that at the end of the study, the changes in the average number of regular and additional medications between the Paro intervention and the control groups were not statistically significant. Contrastingly, the third study (Petersen et al., 2017) reported a significant decrease in the dosage of behavioural (*p* = 0.0009) and pain (*p* = 0.005) medications in the Paro group at post‐intervention compared to control group, but no effect on the dosage of sleep medication (*p* = 0.955) or depression medication (*p* = 0.083).

#### 3.10.8 Apathy

The effect of Paro on apathy was investigated by two studies in residents with dementia (Moyle et al., 2013; Valenti Soler et al., 2015). Pooling of the data was not possible. In comparison with usual care/control, Moyle et al. (2013) reported the effect of Paro on apathy as clinically insignificant, whilst Valenti Soler and colleagues (Valenti Soler et al., 2015) found an improvement, albeit small (*p* = 0.049).

#### 3.10.9 Sleep

The effect of Paro on sleep was investigated by two studies (Moyle et al., 2018b; Thodberg, Sørensen, Videbech, et al., 2016). In both of the studies, Paro was not found to have an effect on sleep, either in terms of sleep patterns (Moyle et al., 2018b) or sleep efficiency (Thodberg, Sørensen, Videbech, et al., 2016).

### 3.11 Overarching synthesis

Figure 1c presents the final iteration of the logic model; thick lines around components indicate where quantitative evidence is available, and where these lines are yellow, statistically significant benefits were reported.

There is overlap between the quantitative and qualitative evidence bases, but also some key differences. The quantitative research focusses on measuring clinical outcomes—with most attention being on measuring the symptoms of BPSD (including agitation, depression and anxiety) and the consequent impact on medication (which the qualitative studies did not explore). However, this is only one component of the model. Impact on staff and relatives, explored in the qualitative research, was not measured in the quantitative research but are important considerations for implementation in care homes. Whilst quantitative studies did explore aspects of engagement, the qualitative evidence synthesis expands this to provide rich detail about how people interact with the robopet and others in the care home (including other residents and care staff). This function of the robopet as a catalyst for communication, connectivity and interaction comes through strongly in the qualitative evidence synthesis, which also shows how contact with the robopet stimulated this through, for example, reminiscence. There were also some negative responses seen in the qualitative evidence, such as the robopets (particularly CuDDler) being perceived by some as potentially infantilising. Conversely, some residents were reported as caring too much for the robopet, potentially increasing anxiety, including around not wanting to share with others.

Alleviating loneliness, identified as important in the qualitative evidence synthesis, was not statistically significant in the pooled analysis in the quantitative review, despite both studies reporting beneficial changes in loneliness. This may be a result of the use of the University of California, Los Angeles (UCLA) loneliness scale. The utility of this scale has been questioned due to its weak theoretical foundation that conceptualises loneliness as a uni‐dimensional concept and the continuous nature of the scale which determines a point that distinguishes lonely from non‐lonely (Victor, 2012). It is also not clear how large a decrease in mean UCLA loneliness score is required to improve the quality of life of an older person.

There was no statistically significant evidence from meta‐analysis on the effects of robopets on other aspects of physical and mental well‐being including depression or quality of life. The qualitative evidence synthesis shows, however, that there is a wide range of responses to robopets, with some residents very keen and others not at all interested. In measuring average impacts, particularly where there are small sample sizes, quantitative research may mask these extremes of response. Impact on sleep was measured in the quantitative review but not reported as an issue in the qualitative evidence synthesis.

## 4 DISCUSSION

This is the first systematic review to bring together qualitative and quantitative evidence of the experiences and effects of robopets for older adults in residential care. Whilst there have been reviews of socially assistive robots or companion robots in older adult care (Bemelmans et al., 2012; Kachouie et al., 2014; Mordoch et al., 2013; Pu et al., 2019), none to date have solely focussed on robopets (animal or pet‐like companion robots), nor on solely on residential care. The qualitative evidence synthesis provides rich detail about the nature of interactions between robopets, residents, staff and family members and describes positive experiences on resident loneliness, depression and quality of life. There was evidence of a reduction in agitation from the meta‐analysis of quantitative research, and the narrative synthesis of quantitative evidence supported findings from the qualitative evidence synthesis of increased interaction and engagement. This could potentially be a mechanism for the observed reductions in agitation and loneliness. There was no statistically significant evidence from meta‐analysis on the effects of robopets on other aspects of physical and mental well‐being, such as depression or quality of life. The effectiveness findings align with those of Pu and colleagues, whose review of social robots (including animal‐like robots) for older adults across all care settings suggested positive impacts on agitation, anxiety and quality of life for older adults but no statistical significance in their meta‐analysis (Pu et al., 2019).

Variation in the nature of robopet “interventions” is important to note and makes unequivocal conclusions on the benefit, or otherwise, difficult to reach with the current state of evidence. There were also a wide range of comparator groups in the studies. Prior research has highlighted how different individual and contextual factors may influence how people respond and interact with robots, in particular that one‐to‐one interaction may be more beneficial than group interactions (Liang et al., 2017). The importance of tailoring and targeting interaction with the robopet to the individual has also been highlighted previously (Bemelmans, Gelderblom, Jonker, & de Witte, 2015, 2016). Care home staff may also require appropriate training and support to enhance the positive impact of the robopet. Indeed, informing caregivers and family members about the purpose and nature of the proposed intervention may help alleviate scepticism and resistance (Bemelmans, Gelderblom, Jonker, & Witte, 2016).

Some of the robopets were very expensive, and this may be prohibitive for some care homes, although as the technology becomes more common, prices may be reduced. The qualitative evidence synthesis also suggests that robopets may not be for everyone, and could annoy or bore some residents, or even cause some to become over‐attached—effects that were not captured in the quantitative evidence, but which have been highlighted by other researchers (Bemelmans et al., 2012; Vandemeulebroucke et al., 2018). Resident health may also impact engagement: with some studies showing lower levels of agitation and higher cognitive functioning to be associated with better responses to robopets (Jones et al., 2018), and others showing lower cognitive functioning to be associated with greater interaction (Thodberg, Sørensen, Videbech, et al., 2016).

Differences between the findings from the qualitative and quantitative evidence may be due to sample sizes in the quantitative research being too small to detect true differences or because the outcomes most important to care home residents were not assessed. Most of the outcomes measured in the quantitative studies were related to symptoms of BPSD, which comprised only a small segment of the overall logic model. It may also be possible that there is an “amalgamation of marginal gains” effect—whilst impact on any single outcome may be small, the overall impact is experienced as beneficial (Richards, 2015).

A strength of this review is that it followed best practice guidelines for both quantitative and qualitative syntheses and was informed by stakeholders. We searched widely for relevant literature and did not limit by date or language, and authors were contacted to provide additional data where necessary. The qualitative and quantitative evidence was brought together through the use of a logic model which developed as the review progressed. We used the logic model as a dynamic tool to refine and actively synthesise the results and bring together findings from both bodies of literature, incorporating stakeholder views in this process. This approach to synthesis was both structured and flexible, allowing for deductive and inductive identification of themes. The review is, however, limited by the quality of the included studies—many of the quantitative studies were small, of short duration and with no follow‐up measurements. In addition, the blinding of participants was often not possible as studies’ aims were commonly disclosed to participants in order to inform consent. Another limitation of the research is the appropriateness of outcome measures. Qualitative research included in the review was generally of higher quality, although few studies were explicitly aligned to a theoretical perspective for their work.

### 4.1 Implications for future practice and research

There are some promising findings in this review suggesting that, through increased engagement with the robopet and collective interactions with the robopet, other residents and staff, there may be benefits for people living in care homes. Using the robopet as a catalyst, these interactions may reduce agitation and loneliness. However, it is clear that not all people are likely to respond positively, so consulting with family members about preferences and history with pets is likely to be important. Staff may also need training to ensure that the robopet is used appropriately, including when to use as part of a group activity and when as a one‐to‐one.

No clear picture emerged about whether one type of robopet is better than another—most research has so far been done on one product. It is also not known if there are long‐term impacts of robopets, or whether novelty confers some of the possible benefit. It is possible that the nature of interactions may change from those initially stimulated through curiosity, but whether these reduce impact or simply change it needs to be investigated. In addition, innovations in methods used in quantitative research to capture the nature of engagement and interaction impacts, as well as how comfort, affection and pleasure may be facilitated, would be useful. The qualitative research also identified some potential harms which could also be incorporated in to future quantitative assessments.

## 5 CONCLUSION

This systematic review integrates the evidence from rich qualitative studies with effectiveness evidence from RCTs on the impact and interactions of robopets for older adults in residential care. Together the findings indicate that robopets, for those that engage and interact with them, appear to have the potential to impact favourably on outcomes such as loneliness and agitation. The evidence to date, however, comes from studies of low to moderate quality and is both diverse and complex. Understanding more about their long‐term impact and the implications for implementation is required before robopets could be considered for routine use with older adults in residential care.

## IMPLICATIONS FOR PRACTICE

- For those that choose to engage with them, robopets have the potential to reduce loneliness and agitation, increase social interactions, as well as provide comfort and pleasure. Interactions are highly varied and influenced by personal histories and the type and characteristics of the robopet.
- Not everyone engages with robopets, and some older adults, families and nursing staff might actively dislike them. Training in how to best use and introduce robopets may help improve resident engagement and staff confidence in using them.
- Whilst robopets should not be considered a replacement for human interaction, there appears to be scope for using them as therapy for agitated or isolated residents.

## CONFLICT OF INTEREST

The authors declare no conflict of interest.

## AUTHOR CONTRIBUTION

RA, JTC and RG conceived the concept of the study and all authors contributed to the design of the study. AB designed the searches. RA, NO, PM, BW and AB screened and data extracted the literature. RA and NO carried out the data syntheses. RA and NO drafted the manuscript, and all authors commented on subsequent drafts and contributed to the discussion and implications.
