# Supplementary material for: How do “robopets” impact the health and well‐being of residents in care homes? A systematic review of qualitative and quantitative evidence
Source: Int J Older People Nurs. 2019 May 9;14(3):e12239. doi: 10.1111/opn.12239 (PMC6766882; doi:10.1111/opn.12239)
Supplement: Supplementary file 1 [file OPN-14-na-s001.docx]

Supplementary Figure 1 Example of MEDLINE search strategy

Figure 1 Database: Ovid MEDLINE(R) Epub Ahead of Print, In-Process & Other Non-Indexed Citations, Ovid MEDLINE(R) Daily and Ovid MEDLINE(R) <1946 to Present>

Search Strategy:

--------------------------------------------------------------------------------

1 (Animal* or pet*).tw. (1101436)

2 (cat or cats).tw. (129838)

3 (dog or dogs).tw. (196858)

4 canine*.tw. (78367)

5 bird*.tw. (64955)

6 robo*.tw. (30516)

7 exp Cats/ (132552)

8 exp Dogs/ (314801)

9 exp Fishes/ (162461)

10 exp Bonding, Human-Pet/ (1683)

11 (fish or fishes).tw. (145492)

12 exp Animal Assisted Therapy/ (354)

13 1 or 2 or 3 or 4 or 5 or 6 or 7 or 8 or 9 or 10 or 11 or 12 (1820437)

14 ((Nursing or geriatric*) adj2 home*).tw. (28784)

15 ((Nursing or geriatric*) adj2 unit*).tw. (3572)

16 ((Nursing or geriatric*) adj2 facilit*).tw. (4061)

17 ((older or senior* or elder* or dement* or Alzheimer*) adj2 institut*).tw. (3111)

18 ((older or senior* or elder* or dement* or Alzheimer*) adj2 resident*).tw. (5396)

19 ((older or senior* or elder* or dement* or Alzheimer*) adj2 unit*).tw. (974)

20 ((older or senior* or elder* or dement* or Alzheimer*) adj2 facilit*).tw. (722)

21 ((older or senior* or elder* or dement* or Alzheimer*) adj2 home*).tw. (3898)

22 14 or 15 or 16 or 17 or 18 or 19 or 20 or 21 (44442)

23 (Care adj2 home*).tw. (24821)

24 ((Long-term or resident*) adj2 care).tw. (24797)

25 (Institut* adj2 (care* or resident* or unit* or facilit* or home*)).tw. (11402)

26 23 or 24 or 25 (56873)

27 (Old* adj2 (people* or person* or adult*or resident*)).tw. (37505)

28 (elder* adj2 (people* or person* or adult*or resident*)).tw. (25368)

29 (geriatric* adj2 (people* or person* or adult*or resident*)).tw. (187)

30 (senior* adj2 (people* or person* or adult*or resident*)).tw. (256)

31 27 or 28 or 29 or 30 (60907)

32 exp Aged/ (2744030)

33 31 or 32 (2759714)

34 26 and 33 (23114)

35 exp Home Nursing/ (9385)

36 exp Homes for the Aged/ (12645)

37 22 or 34 or 35 or 36 (73955)

38 13 and 37 (612)

***************************
