# Supplementary material for: How do “robopets” impact the health and well‐being of residents in care homes? A systematic review of qualitative and quantitative evidence
Source: Int J Older People Nurs. 2019 May 9;14(3):e12239. doi: 10.1111/opn.12239 (PMC6766882; doi:10.1111/opn.12239)
Supplement: Supplementary file 2 [file OPN-14-na-s002.docx]

Supplementary Figure 2 Risk of bias table for included RCTs


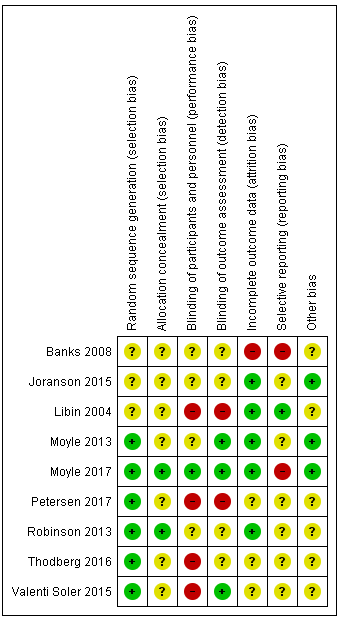


+ low risk of bias, ? unclear risk of bias, - high risk of bias
