# Supplementary material for: How do “robopets” impact the health and well‐being of residents in care homes? A systematic review of qualitative and quantitative evidence
Source: Int J Older People Nurs. 2019 May 9;14(3):e12239. doi: 10.1111/opn.12239 (PMC6766882; doi:10.1111/opn.12239)
Supplement: Supplementary file 3 [file OPN-14-na-s003.docx]

**Table S1. ENTREQ Statement (Enhancing the transparency in reporting the synthesis of qualitative research)**

| **Item** | **Guide and description** | **Page no** |
| --- | --- | --- |
| **1. Aim** | To bring together the evidence of the experiences of staff, residents and family members of interacting with robopets, and the effects of robopets on the health and wellbeing of older people living in care homes. |  |
| **2. Synthesis methodology** | A logic model framework synthesis approach was used to synthesise the qualitative studies. This approach was chosen as it provided an initial framework of themes against which to code the extracted data, but still allowed for themes to be iteratively refined, expanded, created or removed. |  |
| **3. Approach to searching** | Pre-planned, comprehensive search strategy to seek all available studies in the published literature according to a pre-planned, online PROSPERO protocol (CRD42017081794). |  |
| **4. Inclusion criteria** | Phenomenon of interest: Views, experiences and perceptions of interacting with robotic animals of older people in care homes, their families and carers, and care home staff.  Population: Older people resident in care homes, their families and carers, and care home staff.  Language: English language only.  Year: No exclusion based on year of publication.  Types of studies: Qualitative studies using recognised methods of qualitative data collection and data analysis. |  |
| **5. Data sources** | Electronic databases: MEDLINE, EMBASE, PsycINFO, SPP (via OvidSP), CINAHL, AgeLine (via EBSCOhost), CDSR, CENTRAL, DARE (via Wiley Online, Cochrane Library), ASSIA (ProQuest), Web of Science Core Collection, SCOPUS and ProQuest Dissertations and Thesis Global.  Supplementary methods: forward and backward citation chasing of each included article was performed.  Last search April 2017.  An exhaustive search of the literature was completed. |  |
| **6. Electronic search strategy** | Search strategy is described in detail in Figure S1. |  |
| **7. Study screening methods** | Two reviewers (RA, NO or RW) independently screened titles and abstracts against eligibility criteria. The full text of articles initially considered as meeting the inclusion criteria were retrieved and the eligibility criteria applied in the same way. Discrepancies at both stages were discussed and resolved with another reviewer (JTC) where necessary. |  |
| **8. Study characteristics** | Details of the study characteristics are provided in Table 1. |  |
| **9. Study selection results** | Figure 2 outlines the study selection process in a PRISMA* flow diagram. |  |
| **10. Rationale for appraisal** | The purpose of the quality appraisal was to critically appraise the qualitative studies. |  |
| **11. Appraisal items** | The Wallace criteria was used to critically appraise the qualitative studies. |  |
| **12. Appraisal process** | The quality appraisal was conducted independently by two reviewers (RA & NO) and consensus reached by discussion. |  |
| **13. Appraisal results** | The quality appraisal results are available in Table S3. We did not exclude any articles on the basis of quality as we believed that all studies may contribute some important insights to the phenomenon of interest. |  |
| **14. Data extraction** | All content in the results, discussion and conclusion sections of the included papers were considered as data for analysis. These data were extracted into a bespoke data extraction form derived from the logic model (see Figure 1a for the logic model). Data extraction was completed by two reviewers independently.  Information extracted: date of publication, country of conduct, study description, participants, setting, study aim and context, data collection and data analysis were extracted from the included studies and are presented in Table 1. |  |
| **15. Software** | Not used. |  |
| **16. Number of reviewers** | Two reviewers (RA & NO) read all the included studies in detail and independently extracted data from the papers, meeting to discuss the findings, and reach a consensus. Other reviewers (RG, JTC) were involved in the synthesis. |  |
| **17. Coding** | The logic model framework was used for initial coding of the data; data that could not be accommodated within the framework were coded line by line to search for new themes. |  |
| **18. Study comparison** | The data were compared across studies and two reviewers (RA, NO) discussed whether all components of the model were observed in the data and whether any new components were evident in the data that were not part of the initial model (Figure 1a). These reviewers refined the logic model and produced a second iteration (see Figure 1b) which included both modified and new elements that had not been anticipated in the first iteration. |  |
| **19. Derivation of themes** | The process of developing the themes was both deductive and inductive: initially, we used the logic model to identify *a priori* themes and then we created new themes inductively. |  |
| **20. Quotations** | Direct quotes from the participants – residents, family members and care staff - are presented in the Results section of the manuscript and in more detail in Table S5. |  |
| **21. Synthesis outputs** | The qualitative evidence and quantitative evidence were brought together in an overarching synthesis and in a final iteration of the logic model (see Figure 1c). There is overlap between the qualitative and quantitative evidence bases but also some key differences. |  |

*PRISMA – Preferred Reported Items for Systematic Reviews and Meta-Analyses.
