# Supplementary material for: How do “robopets” impact the health and well‐being of residents in care homes? A systematic review of qualitative and quantitative evidence
Source: Int J Older People Nurs. 2019 May 9;14(3):e12239. doi: 10.1111/opn.12239 (PMC6766882; doi:10.1111/opn.12239)
Supplement: Supplementary file 5 [file OPN-14-na-s005.docx]

**Table S3. Quality Appraisal of Included Studies**

| **Author(s)** | **Is the research question clear?** | **Perspective of author clear?** | **Perspective influenced the study design?** | **Is the study design appropriate?** | **Is the context adequately described?** | **Sample adequate to explore range of subjects/settings?** | **Sample drawn from appropriate population?** | **Data collection adequately described?** | **Data collection rigorously conducted?** | **Data analysis rigorously conducted?** | **Findings substantiated/ limitations considered?** | **Claims to generalizability follow from data?** | **Ethical**  **issues**  **addresse*d*?** |
| --- | --- | --- | --- | --- | --- | --- | --- | --- | --- | --- | --- | --- | --- |
| Birks et al 2016 | Y | N | N | Y | Y | N | Y | CT | Y | Y | Y  Y | Y | Y |
| Chang et al 2013 | N | N | N | Y | Y | Y | Y | CT | CT | CT | N  N | CT | Y |
| Chang & 2015 | Y | Y | Y | Y | Y | Y | Y | Y | Y | Y | Y  Y | Y | CT |
| Giusti & Marti 2006 | Y | Y | Y | Y | Y | Y | Y | Y | Y | CT | Y  N | Y | CT |
| Gustaffson et al 2015 | Y | N | N | Y | Y | Y | Y | Y | Y | Y | Y  Y | Y | Y |
| Iacono & Marti 2016 | Y | Y | Y | Y | Y | Y | Y | Y | Y | Y | Y  N | Y | Y |
| Jung et al 2017 | Y | N | N | Y | Y | Y | Y | Y | Y | Y | Y  Y | Y | Y |
| Moyle et al 2016 | Y | N | N | Y | Y | Y | Y | Y | Y | Y | Y  Y | Y | Y |
| Moyle et al 2017a | Y | N | N | Y | Y | Y | Y | Y | Y | Y | Y  N | Y | Y |
| Moyle et al 2018a | Y | N | N | Y | Y | Y | Y | Y | Y | Y | Y  Y | Y | Y |
| Moyle et al 2019 | Y | N | N | Y | Y | Y | Y | Y | Y | Y | Y  Y | Y | Y |
| Niemela et al 2016 | Y | N | N | Y | Y | Y | Y | CT | CT | CT | Y  N | Y | N |
| Pfadenhauer & Dukat 2015 | Y | Y | Y | Y | Y | CT | Y | CT | CT | CT | Y  Y | CT | N |
| Robinson et al 2013 | Y | N | N | Y | Y | Y | Y | Y | Y | CT | Y  Y | Y | Y |
