# Supplementary material for: How do “robopets” impact the health and well‐being of residents in care homes? A systematic review of qualitative and quantitative evidence
Source: Int J Older People Nurs. 2019 May 9;14(3):e12239. doi: 10.1111/opn.12239 (PMC6766882; doi:10.1111/opn.12239)
Supplement: Supplementary file 6 [file OPN-14-na-s006.docx]

**Table S4. Summary of Framework Themes**

| **Authors** | **Robopet** | | | | **Older Person/Resident** | | | **Person-Person and Staff** | |
| --- | --- | --- | --- | --- | --- | --- | --- | --- | --- |
|  | **Responsiveness** | **Entertainment/**  **Stimulation** | **Something to care for** | **Confiding/**  **Opportunity to communicate** | **Reminiscence** | **Identity/**  **Belonging** | **Sensory** | **Trigger for conversation** | **Social Contact** |
| Birks 2016 | **✓** | **✓** |  | **✓** | **✓** | **✓** | **✓** |  | **✓** |
| Chang 2013 |  |  |  |  |  |  |  |  | **✓** |
| Chang 2015 | **✓** | **✓** | **✓** | **✓** |  | **✓** |  | **✓** | **✓** |
| Giusti 2006 | **✓** | **✓** | **✓** |  |  |  | **✓** |  | **✓** |
| Gustaffson 2015 | **✓** | **✓** | **✓** |  | **✓** |  | **✓** |  | **✓** |
| Iacono 2016 | **✓** | **✓** | **✓** |  |  |  | **✓** |  |  |
| Jung et al 2017 | **✓** | **✓** |  |  |  |  | **✓** |  | **✓** |
| Moyle et al 2016 | **✓** |  |  |  | **✓** |  |  | **✓** | **✓** |
| Moyle et al 2017a | **✓** |  | **✓** |  | **✓** |  |  |  | **✓** |
| Moyle et al 2018a | **✓** |  | **✓** |  |  | **✓** |  |  | **✓** |
| Moyle et al 2019 |  | **✓** |  |  |  |  |  |  |  |
| Niemela et al 2016 |  |  |  |  |  |  |  |  |  |
| Pfadenhauer 2015 |  |  |  |  | **✓** |  |  |  | **✓** |
| Robinson et al 2013 | **✓** | **✓** | **✓** | **✓** |  |  | **✓** | **✓** | **✓** |

**Table S4. Summary of Framework Themes**

| **Authors** | **Resident Quality of Life** | | | | **Staff** | | **Family** | **Barriers for Residents** | | | **Barriers for Staff** | | | | | |
| --- | --- | --- | --- | --- | --- | --- | --- | --- | --- | --- | --- | --- | --- | --- | --- | --- |
|  | **Loneliness** | **BPSD** | **Comfort**  **And Safety** | **Pleasure and Joy** | **Usefulness** | **Therapeutic Tool** | **Appreciate**  **value** | **Sensory dislike** | **Toy-like** | **Care too much** | **Expensive** | **Hygiene** | **Heavy** | **Ethics** | **Training** | **Not for all** |
| Birks 2016 | **✓** | **✓** | **✓** | **✓** | **✓** | **✓** | **✓** |  |  |  | **✓** |  |  |  |  | **✓** |
| Chang 2013 |  | **✓** | **✓** |  |  |  |  |  |  |  |  |  |  |  |  |  |
| Chang 2015 |  |  | **✓** | **✓** | **✓** | **✓** |  |  |  |  |  |  |  |  |  |  |
| Giusti 2006 |  |  |  |  |  |  |  |  |  |  |  |  |  |  |  |  |
| Gustaffson 2015 | **✓** | **✓** | **✓** | **✓** | **✓** | **✓** | **✓** |  |  | **✓** |  |  |  |  |  |  |
| Iacono 2016 |  |  |  |  |  | **✓** |  |  |  |  |  |  |  |  |  |  |
| Jung et al 2017 | **✓** | **✓** |  |  |  | **✓** |  |  |  |  | **✓** |  |  | **✓** | **✓** | **✓** |
| Moyle et al 2016 | **✓** | **✓** |  | **✓** |  |  |  | **✓** | **✓** |  |  |  | **✓** | **✓** |  |  |
| Moyle et al 2017a |  | **✓** |  | **✓** |  |  | **✓** |  |  | **✓** |  |  |  |  | **✓** |  |
| Moyle et al 2018a | **✓** | **✓** | **✓** | **✓** | **✓** |  |  |  |  |  | **✓** | **✓** | **✓** |  | **✓** | **✓** |
| Moyle et al 2019 | **✓** | **✓** |  |  |  |  |  |  |  |  | **✓** |  |  |  |  | **✓** |
| Niemela et al 2016 |  |  |  |  |  | **✓** |  |  |  |  | **✓** | **✓** |  | **✓** | **✓** |  |
| Pfadenhauer 2015 |  |  |  |  |  |  |  |  |  |  |  |  |  |  |  |  |
| Robinson et al 2013 | **✓** |  | **✓** | **✓** |  |  |  | **✓** | **✓** |  |  |  | **✓** |  |  | **✓** |
