# Supplementary material for: How do “robopets” impact the health and well‐being of residents in care homes? A systematic review of qualitative and quantitative evidence
Source: Int J Older People Nurs. 2019 May 9;14(3):e12239. doi: 10.1111/opn.12239 (PMC6766882; doi:10.1111/opn.12239)
Supplement: Supplementary file 7 [file OPN-14-na-s007.docx]

**Table S5. Qualitative findings with illustrative quotes**

| **Component** | **Theme** | **Sub-theme** |  | **Illustrative Quotation** |
| --- | --- | --- | --- | --- |
| **Resident Robopet Engagement** | Responsiveness | Emotional (+) |  | “J’s facial expressions (smiles and laughter) suggested she found the interaction with CuDDler positive…J displayed pleasure when CuDDler responded to her touch”^(Moyle et al 2016)^ (p. 151, reviewer edit). |
|  |  | Behavioural (+) |  | Therapist 3 - “on entering the room I noticed her looking forlorn, distant and looking into space…she spotted George (Paro) in my arms and *her whole body language changed* immediately by showing exuberance, verve in her movements and in her face. She immediately *held out her arms to hold George*” ^(Birks et al 2016)^ (Log entry, p. 3, reviewer emphasis). |
|  |  | Visual (+) |  | Therapist 1 – “There was one lady…she’s a loner and she stays in her room at all times. Having…Sally placed in her arms, it just reminded her of her baby. She just *opened up and her face just lit up*…it was a just powerful moment and…it was just beautiful just to be there and to experience it” ^(Birks et al 2016)^ (p. 4, reviewer emphasis). |
|  |  | Verbal (+) |  | “’G’ always praised CuDDler as ‘beautiful’, ‘gorgeous’, ‘sweet’, and ‘lovely bear’. She also called CuDDler a ‘sweetheart’ and ‘darling’”^(Moyle et al 2016)^ (p. 156, author quote). |
|  |  | Indifference |  | Resident – “I didn’t particularly feel anything towards it. I can’t go gushy over a soft animal like some of the others do”^(Robinson et al 2016)^ (p. 108). |
|  |  | Not interested |  | “‘A’ refused to hold CuDDler on her lap and said she was ‘too old’ to interact with CuDDler”^(Moyle et al 2016)^ (p. 152). |
|  |  | Verbal & Behavioural (-) |  | “…When the RA [research assistant] attempted to give her Paro, Margaret was perched on the side of the bed, holding a piece of paper. She was *verbally aggressive*, standing up off the bed and saying ‘well I can’t because I’ve got to go and see my shoes’ and in a *raised voice with hand gestures*, ‘I don’t want it’…In week 5 she refused to let the RA into her room even before Paro was shown, repeating as she did in week 1, ‘No I have to go and see my shoes.’ In week 10, whilst walking past Paro in the corridor, Margaret did stroke it under the chin as she passed. However, she was *not interested in holding it when asked by RA* and she *did not actively look at Paro* and instead, continued to walk around the facility”^(Moyle et al 2019)^ (p. 3, reviewer edit and emphasis). |
|  |  | Variability | Time | Therapist 1 - “There have been a couple of residents that have been a *little wary* of George and Sally. *I’ve gone back to those residents, and some of them have actually come forward and stroked George or Sally*. The others have dismissed me straight away; don’t want to have anything to do with it”^(Birks et al 2016)^ (p. 2, reviewer emphasis). |
|  |  |  | Dementia | Therapist 1 – “I think the [residents with] dementia react better. They *smile a lot more*, or they *frown*, or their *eyes sparkle*” ^(Birks et al 2016)^ (p. 3, reviewer emphasis). |
|  | Entertainment and Stimulation |  |  | Daughter – “She’s always been bored in there [nursing home]…So, she likes interacting with it because it gave her something to do”^(Moyle et al 2017a)^ (FD7, p. 5-6). |
|  |  | Humour & play |  | Therapist 2 – *“They were all excited. It was a very noisy atmosphere and everyone was talking at the same time, laughing, giggling and elbowing each other trying to come up some rhyming words* [sic]. Some residents [came] up with funny words and some [were] not suitable, but it sounded very funny and *they all laughed*…Residents were *using their brains* it stimulation them [sic]. It was also good social interaction and a good laugh. Until next time, I am planning to record the song on CD when completed. [It’s a] good sense of achievement for the residents”^(Birks et al 2016)^ (p. 4, Log entry, reviewer emphasis). |
|  |  | Curiosity |  | “Resident E *showed great interest in Paro since the first time she saw it*. She approached Paro spontaneously and had a long talk with it. She interacted with Paro in *exploratory* as well as reflective ways. She *asked Paro* instead of the staff and researchers, *many questions to figure out what it is* (e.g. ‘Who are you?’ ‘Are you a cat or a dog?’), its behaviour and history (e.g. ‘Are you looking at me?’ ‘Are you sleepy?’ ‘Did they mistreat you?’), and its emotions (e.g. ‘Are you mad at me?’ ‘Are you sad?’ ‘You want me to leave?’”^(Chang et al 2015)^ (p. 348, reviewer emphasis). |
|  |  | Bored |  | Resident – “I got bored with it. I would rather do most other things”^(Robinson et al 2016)^ (p. 108). |
|  | Something to care for |  |  | Staff - “…when I saw them interacting with it…you saw their loving personality came back. They knew that *they needed to look after this thing* that was being handed to them”^(Moyle et al 2018a)^ (Endorsed Enrolled Nurse 1, p. 3 reviewer emphasis). |
|  | Confiding/  opportunity to communicate |  |  | Therapist 3 – “As soon as he viewed George his eyes opened wide and a huge smile appeared on his face…he placed his water on the bed side table and opened his arms ready to take hold of him and *commenced talking to George as if he was continuing his conversation from the last visit with Sally*. He commenced with, ‘What have you been doing with yourself?’ *Conversation flowed as he held George*”^(Birks et al 2016)^ (Log entry p. 3-4, reviewer edit and emphasis). |
| **Resident** | Reminiscence |  |  | Professional caregiver – “[the participant] began *to talk about animals he had had* and…that he had worked with and he said he liked being out in the forest looking at the animals and just sitting and enjoying nature. ‘I miss that a little bit,’ he said…He had not talked about it like that before; it was the first time that happened like that”^(Gustafsson et al 2016)^ (p. 52, (reviewer emphasis). |
|  | Sensory experience |  |  | Therapist 1 – “…and her eyes just sparkle when I walk in and put George or Sally [Paro] on the bed table, because *she can touch and stroke* [him] and all that that”^(Birks et al 2016)^ (p. 3, reviewer emphasis). |
|  | Identity/  Belonging |  |  | “I think it would give them…a sense of being loved as well because a lot of dementia residents, the families don’t cope well with the dementia, so they don’t visit as often”^(Moyle et al 2018a)^ (Endorsed Enrolled Nurse 1, p. 3). |
|  |  | Gender |  | “…the wife noticed Paro and excitedly pointed it out to her husband. The husband just commented he had seen it before and did not show much interest. In two other cases, the female partner[sic] was excited about Paro and brought their male companion back to the area to show the robot to them. On both occasions, the man stayed in the hallway and looked at their companion petting and talking to Paro from a distance without interacting directly with the robot”^(Chang et al 2015)^ (p. 347, reviewer edit). |
| **Person-Person** | Trigger for conversation |  |  | …In one visit, Resident D found Paro and petted it while observing the researcher’s reaction to her interaction and having a conversation with the researcher about it. *Once the researchers stopped talking to her, she quickly lost interest in Paro* and paid attention to other things. In another visit, Resident D started off by ignoring Paro and watching TV. When two staff approached Paro and her, and talked to her about how cute it was, she started petting the robot and *happily conversed with the staff*. Shortly after the staff left, she lost interest in Paro again. For Resident D, Paro functioned as *a tool to generate desired social interaction with other people”^(^*^Chang et al 2015)^ (p. 348, reviewer emphasis). |
|  | Social contact |  |  | Son - “…now we have something to talk about-the robot cat! Conversations about the weather and the meals are so meaningless; *the robot cat has given us meaning in our communication*”^(Gustafsson et al 2016)^ (p. 52, reviewer emphasis). |
| **Resident Quality of Life** | Reduced  loneliness |  |  | Resident – He [Paro] is a good companion when you are lonely”^(Robinson et al 2016)^(p. 108). |
|  | Increased pleasure and joy |  |  | Resident – “Very happy, relaxed. It was a pleasure to be with her [Paro]”^(Robinson et al 2016)^ (p. 108). |
|  | Increased comfort and safety |  |  | Professional caregiver – “Well, [the participant] fell asleep with the robotic cat on his chest, in his arms, and we consider it as reducing his loneliness. He is nearly blind and, with the robot cat, he experiences *feelings of comfort and security*”^(Gustafsson et al 2016)^(p. 52-3, reviewer emphasis). |
|  | BPSD |  |  | Therapist 3 – “I heard the resident yelling….as soon as he saw me he stopped yelling and beckoned for me to come over with George [Paro], He spent the rest of the time...touching and observing the movements of George. After leaving, I listened and he did not commence his usual yelling for help. Also I returned 20 minutes later to listen for yelling and did not hear the resident verbalising”^(Birks et al 2016)^ (Log entry, p. 4). |
| **Staff** | Usefulness |  |  | Staff – “Very convenient and it’s there as a wonderful support…when the residents are going through this agitation or this distressing period in their day you can bring out the robotic animal…”^(Moyle et al 2018a)^ (Activities Coordinator 3, p. 3). |
|  |  |  |  | Therapist 2 – “It’s just the reaction…They’re in the moment on the spot…it *just gives you joy*, you can see their faces”^(Birks et al 2016)^ (p. 5, reviewer emphasis). |
|  | Therapeutic Tool |  |  | Therapist 1 – “…it is a therapeutic tool, but still I don’t think it’s for everybody”^(Birks et al 2016)^ (p. 3). |
| **Family** | Appreciation of Value (+) |  |  | Therapist 1 - “I took George this morning to see a resident, her daughter came to me the other day and said…’I won’t be visiting mum…on Thursday, could you please take George or Sally up to her?’”^(Birks et al 2016)^ (p. 4). |
|  | Appreciation of Value (-) |  |  | Therapist 3 – “…somebody said to me, a family member said ‘oh, I’ve seen somebody carrying that around, they’re *like a complete idiot*”^(Birks et al 2016)^ (p. 3, reviewer emphasis). |
| **Challenges - Resident** | Sensory Dislike |  |  | “’E’ described CuDDLer as ‘too mechanical’ and ‘looks a bit dead’. She also found it to be ‘very heavy and it is difficult to hold him up’. ‘S’ also had similar concerns and said CuDDLer was too heavy to hold and preferred CuDDLer to sit on the table rather than in her lap.”^(Moyle et al 2016)^ (p. 153) |
|  | Toy–like |  |  | “’S’ stated that CuDDler was a ‘toy for children’ and she was ‘too old for it’. Throughout the interview, ‘S’ was consistent in her reply that she ‘was too old for toys like that. I don’t think it has a lot of use for adults. They might think it’s too much of a toy for adults.’…She thought it did not make her feel any different and it was ‘a bit of a waste of money’”^(Moyle et al 2016)^ (p. 153, author quote, reviewer edit). |
|  | Care too much |  |  | Betty was sitting watching television…when Paro was introduced during a session in week 5…Betty was immediately engaged with Paro, continuously looking at Paro and stroking his back. Another resident, Mary, sat to her right, and *also showed an interest in Paro by persistently leaning over to stroke Paro whilst it sat on Betty’s lap*. *Betty permitted this interaction initially but, after approximately six minutes, she became agitated and tried to push Mary’s hand away, saying ‘I don’t think you should be doing that’ and then, a minute later, ‘I don’t like it’ when Mary tried to stroke Paro…* When the session was brought to an end by the RA, Betty became visibly distressed, leaning forward from her chair and saying to the RA ‘I’m not liking you’ and then ‘no why do you have to take him away while he’s here…he’s happy with me’^(Moyle et al 2019)^ (p. 5, reviewer edit, reviewer emphasis). |
| **Challenges -Staff** | Expensive |  |  | “From a facility point of view, I don’t think we could actually afford a seal”^(Moyle et al 2018a)^ (Facilities Manager 1, p. 3). |
