# Supplementary material for: How do “robopets” impact the health and well‐being of residents in care homes? A systematic review of qualitative and quantitative evidence
Source: Int J Older People Nurs. 2019 May 9;14(3):e12239. doi: 10.1111/opn.12239 (PMC6766882; doi:10.1111/opn.12239)
Supplement: Supplementary file 8 [file OPN-14-na-s008.docx]

Supplementary Table 6 Summary of key outcome data from the RCT studies

| **Outcome**  Study ID | **Scale/Tool** | **Comparator groups and sample size**  Intervention(I), Control (C), Other (Oth) | **Post Intervention scores or Change scores**  Mean (SD) | **Interpretation and significance (as reported by authors)** |
| --- | --- | --- | --- | --- |
| *Loneliness* |  |  |  |  |
| Banks 2008 | UCLA | Aibo vs real dog vs usual care (control)  (I:12, Oth:13, C:13) | 41 (8.58) vs 47.4 (4.25) vs 44.6 (8.25) ** | Control group statistically more lonely than AIBO (P<0.05) and Dog (P<0.05) group, but no statistically significant difference between AIBO and Dog groups |
| Robinson 2013 | UCLA | Paro vs usual care (control)  (I:17, C:17) | 32.23 (9.92) vs 33.93 (8.52) | P = 0.03, PARO group statistically less lonely than control |
| *Agitation* |  |  |  |  |
| Joranson 2015 | BARS | Paro vs usual care (control)  (I:27, C:26) | 20.2 (10.1) vs 24.7 (14) | Post intervention P=0.098 (post compared to baseline), not significant. Follow up p=0.048 (follow up compared to baseline), statistically significant |
| Moyle 2017b | CMAI measured | Paro vs plush toy vs routine care (control)  (I:72, Oth:70, C:67) | 27.86 (11.43) vs 29.06 (10.55) vs 31.38 (11.3) ** | Paro vs control p=0.343, not significant, Paro vs plush toy p=0.684, not significant |
| Moyle 2017b | CMAI  observed | Paro vs plush toy vs routine care (control)  (I:138, Oth:140, C:137) | NA | Significant change in time reported for Paro vs control [3.33 (5.79-0.86), p=0.008] but not Paro vs Plush Toy [1.28 (4.21 –1.66), p=0.393]. |
| *Depression* |  |  |  |  |
| Joranson 2015 | CSSDD | Paro vs usual care (control)  (I:27, C:26) | 8.1 (5.6) vs 7.9 (6.7) | Post intervention p=0.098 (post compared to baseline), not significant. Follow up p=0.028 (follow up compared baseline), statistically significant |
| Peterson 2017 | CSSDD | Paro vs usual care (control)  (I:35, C:26) | Mean change scores:  2.81 ().4) vs 0.78 (0,4) | Greater reduction in depression with Paro compared to control, p = 0.001 |
| Moyle 2013 | GDS | Paro versus reading control  (crossover n=18) | 4.3 (3.5) vs 4.7 (2.9) | Estimate of Cohen’s d effect (0.1- 0.1). change deemed ‘not clinically significant’ and ‘no reliable change’ with reliability change index of 1.29 |
| Robinson 2013 | GDS | Paro vs usual care (control)  (I:17, C:17) | 4.15(2.34) vs 4.0 (2.62) | No significant effect of Paro on depression compared to control (p=0.97) |
| Thodberg 2015 | GDS | Paro vs living dog vs plush cat (control)  (I:35, Oth: 35, C: 30) | Data not normal: medians and interquartile range data available  2 [1; 3] vs 2 [1; 3] vs 2 [1; 4] ** | No effect of Paro on depression compared to control or real dog (F2.82; P>0.05) |
| *Quality Of Life (QoL)* |  |  |  |  |
| Joranson 2016 | QUALID | Paro vs usual care (control)  (I:27, C:26) | 23.4 (7.65) vs 25.3 (10.3) | No effect of Paro on QoL at Post intervention p=0.121 (or at follow-up p=0.117 at 3 months) |
| Valenti-Soler 2015 | QUALID | Paro vs living dog vs usual care (control)  (I:42, O:36, C:32) | 26.75(8.16) vs 24.33 (6.68) vs 24.72 (6.68) | No difference in Qol Scores between Paro and dog (p=0.547) or between Paro and control (p=0.101) |
| Moyle 2013 | QOL-AD | Paro versus reading control  (crossover n=18) | 37.2 (8.2) vs (26.4 (16.8) | Estimate of Cohen’s d effect (0.6 -1.3), reliability change index (4.48), change deemed reliable and significant. |
| Robinson 2013 | QOL-AD | Paro vs usual care (control)  (I:17, C:17) | 32.73 (8.24) vs 31.19 (6.26) | No effect of Paro on QoL for Paro compared to Control, (F=0.22, df=1,28, p=0.64) |
| *Engagement/Interaction* |  |  |  |  |
| Libin 2004 | Direct  Observation  (bespoke tool) | Robotic cat vs plush toy cat (control)  (crossover n=9) | No data available | Paired t-test showed no difference between the two groups |
| Moyle 2017b | Video observation (GoPro) | Paro vs plush toy  (I:138, Oth:140) - not measured for C | Adjusted mean differences:  Behavioural 6.34 (13.45 to -0.77)  Social 1.22 (2.99 to -0.56)  Verbal 3.61 (6.40 to 0.81)  Visual 13.06 (17.05 to 9.06) | Significant increases in verbal (p=0.11) and visual (p<0.001) engagement, but not for behavioural (p=0.0), or social (p=0.170) engagement. |
| Robinson 2013 | Direct  Observation  (bespoke tool) | Paro vs usual care (control)  (I:17, C:17) | Talking to each other: Mann- Whitney U test U=21.00, z=2.35, P=0.02) | Residents talked more to each other in the presence of Paro than compare to control, and a higher percentage of residents talked to each other with Paro compared to control |
| Thodberg 2016 | Direct observation  (bespoke tool) | Paro vs living dog vs plush cat (control)  (I:35, Oth: 35, C: 30) | Conversation (various)  Physical contact (various)  Eye contact (various) | Paro and the dog triggered more interaction than the plush cat, however Paro interaction decreased over time compared to the dog. Cognitive impairment affected interaction. |
| *Anxiety* |  |  |  |  |
| Moyle 2013 | RAID | Paro versus reading control  (crossover n=18) | 9.8 (6.5) vs 7 (6.9) | Estimate of Cohen’s d effect (0.4 to 0.4), reliability change index 3.80, change deemed not reliable or clinically significant. |
| Moyle 2017b | Video observation | Paro vs plush toy  Paro vs routine care (control)  (I:138, Oth:140, C:137) | Adjusted mean difference:  -0.99 (0.04 to -2.02), p=0.060  -0.28 (0.4 to -0.96), p=4.22 | No effect of Paro on anxiety compared to control or plush toy |
| Peterson 2017 | RAID | Paro vs usual care (control)  (I:35, C:26) | 2.5 (0.6) vs 0.55 (0.2) | Paro decreased anxiety compared to control (p=0.003) |
|  |  |  |  |  |
|  |  |  |  |  |
| *Medication* |  |  |  |  |
| Joranson 2015  & 2016 | Medicine overviews | Paro vs usual care (control)  (I:27, C:26) | No raw data reported for groups.  Subgroup analyses psychotropics  Severe: 0.75(0.46) vs 1.67(0.46), p=0.007  Mild/moderate: 0.92(0.95) vs 1.08(0.9), p=0.670 | Changes in regular and additional medication was not significant between groups at any time point. Post hoc sub-group analyses showed significantly lower use of psychotropic drugs recorded for those with more severe dementia, but no effect for mild to moderate dementia. |
| Mervin 2018 | Extracted from medical records | Paro vs plush toy  Paro vs routine care (control)  (I:138, Oth:140, C:137) | No data presented | No significant differences in average no. medications between groups |
| Peterson 2017 | Retrospective & concurrent | Paro vs usual care (control)  (I:35, C:26) | Pain 2.22(0.7) vs 0.26(0.5)  Sleep 0.03(0.6) vs 0(0)  Depression -0.68(0.4) vs 0(0)  Behaviour -2.09 (0.54) vs -0.09(0.09) | Significant reductions in pain (p=0.005) and behaviour (p=0.0009) medications, but not sleep (p=0955) or depression (p=0.083) medications |
| *Apathy* |  |  |  |  |
| Moyle 2013 | AES | Paro versus reading control  (crossover n=18) | 38.7 (13.7) vs 36.5 (13.7) | Estimate of Cohen’s d effect (0.2 to 0.2), reliability change index (7.51), change deemed not reliable or clinical significant |
| Valenti-Soler 2015 | APADEM-NH | Paro vs usual care (control)  (I: 42, C:32) | 44.74 (22.29) vs 44.42 (23.59) | Paro group showed improvement in apathy compared with control group, taking into account baseline scores (p=0.049) |
| *Sleep* |  |  |  |  |
| Moyle 2018b | Sensewear armband | Paro vs plush toy  Paro vs routine care (control)  (I:138, Oth:140, C:137) | −0.46 (0.51 to −1.42), p=0.354  0.58 (0.40 to −1.57), p=0.245 | No evidence that PARO was effective in improving sleep patterns |
| Thodberg 2016 | Actigraph | Paro vs living dog vs plush cat (control)  (I:35, Oth:35, C:30) | Efficiency %: 75(15) vs 79(18) vs 74(17)  Frag index: 63(23) vs 52(32) vs 56(35) | Paro did not affect sleep efficiency or sleep fragmentation compared to control or living dog |

** data obtained from authors

UCLA: University of California Los Angeles Loneliness Scale, BARS: Brief Agitation Rating Scale, CMAI: Cohen-Mansfield Agitation Inventory, CSDD: Cornell Scale for Symptoms of Depression in Dementia, GDS: Geriatric Depression Scale, QUALID: Quality of Life in Late-stage Dementia, QOL-AD: Quality of Life in Alzheimer’s Disease, AES: Apathy Evaluation Scale, APADEM-NH: Apathy Scale for Institutionalized Patients with Dementia Nursing Home version, RAID: Rating Anxiety in Dementia
